# Studies on the Nonalkaloidal Secondary Metabolites of *Hippeastrum vittatum* (L’Her.) Herb. Bulbs

**DOI:** 10.1021/acsomega.2c07886

**Published:** 2023-07-23

**Authors:** Marwa Fathy Khalifa, John Refaat Fahim, Ahmed E. Allam, Mai E. Shoman, Amr El Zawily, Mohamed Salah Kamel, Kuniyoshi Shimizu, Eman Zekry Attia

**Affiliations:** † Department of Pharmacognosy, Faculty of Pharmacy, Minia University, 61519 Minia, Egypt; ‡ Department of Pharmacognosy, Faculty of Pharmacy, Al-Azhar University, 71524 Assiut, Egypt; § Department of Medicinal Chemistry, Faculty of Pharmacy, Minia University, 61519 Minia, Egypt; ∥ Department of Plant and Microbiology, Faculty of Science, Damanhour University, 22511 Damanhour, Egypt; ⊥ Department of Biology, University of Iowa, Iowa City, Iowa 52242-1324, United States; # Department of Agro-Environmental Sciences, Graduate School of Bioresource and Bioenvironmental Sciences, Kyushu University, 744 Motooka, Nishi-ku, 819-0395 Fukuoka, Japan

## Abstract

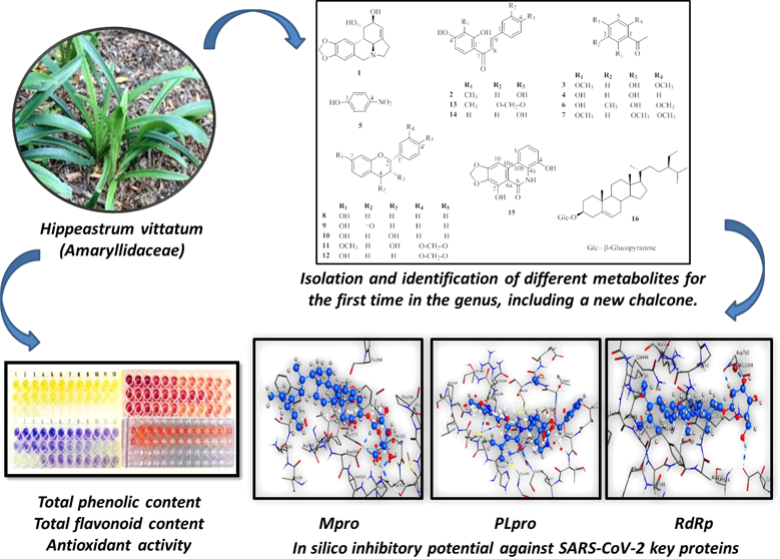

Sixteen chemically varied metabolites were isolated from the bulbs of *Hippeastrum vittatum* (L’Her.) Herb., including eight flavonoids [3′-methyl isoliquiritigenin **(2)**, 7-hydroxyflavan **(8)**, 7-hydroxyflavanone **(9)**, 7-hydroxyflavan-3-ol **(10)**, 7-methoxy-3′,4′-methylenedioxyflavan-3-ol **(11)**, 7-hydroxy-3′,4′-methylenedioxy flavan **(12)**, 2′,4′-dihydroxy-3′-methyl-3,4-methylenedioxychalcone **(13)**, and isoliquiritigenin **(14)**], four acetophenones [2,6-dimethoxy-4-hydroxyacetophenone **(3)**, 2,4-dihydroxyacetophenone **(4)**, 2,4-dihydroxy-6-methoxy-3-methylacetophenone **(6)**, and 2,4,6-trimethoxyacetophenone **(7)**], two alkaloids [lycorine **(1)** and narciprimine **(15)**], one phenol derivative [*p*-nitrophenol **(5)**], and one steroid [β-sitosterol 3-*O*-β-glucopyranoside **(16)**]. Their structures were elucidated by combining one- and two-dimensional NMR and ESI-MS techniques and by comparison with the reported literature data and some authentic samples. Except for lycorine **(1)**, the isolated metabolites were obtained herein for the first time from *Hippeastrum* plants, among which compound **13** was identified as a new chalcone derivative. Additionally, the total phenolic and flavonoid contents of the total ethanol extract and different fractions of the bulbs were determined by the Folin–Ciocalteu and aluminum chloride colorimetric methods, respectively, whereas their antioxidant potential was compared using the phosphomolybdenum and 2,2′-diphenyl-1-picrylhydrazyl (DPPH) free radical scavenging assays. Finally, the binding affinities of compounds **1–16** to some key target proteins of the severe acute respiratory syndrome coronavirus 2 (SARS-CoV-2), namely, main protease (M^pro^), papain-like protease (PLpro), and RNA-dependent RNA polymerase (RdRp), were screened and compared using molecular docking analysis. The possible chemotaxonomic significance of the identified metabolites was also discussed.

## 1 Introduction

Amaryllidoideae is a large subfamily of monocotyledonous flowering plants in the family Amaryllidaceae with about 800 species distributed in 70 genera.^1^ Members of the Amaryllidoideae are bulbous geophytic plants found throughout the tropical and subtropical habitats, with exceptional biodiversity in South America, the Mediterranean region, and South Africa.^2^ These plants have been acknowledged as talented sources of varied phytochemicals owing to their content of a myriad of alkaloidal and nonalkaloidal constituents with praiseworthy biological profiles, including among others, anti-Alzheimer’s, antibacterial, antiparasitic, antitumor, antiviral, hepatoprotective, and immunomodulatory properties.^2−4^ Among these plant species, those of the genus *Hippeastrum* are well-known ornamental icons and multipurpose folk remedies, commonly used for asthma, piles, tumors, and other inflammatory conditions.^5^ To date, *Hippeastrum* plants, including *Hippeastrum vittatum* (L’Her.) Herb., have attracted particular interest, thanks to their abundant Amaryllidaceae alkaloids and notable anticholinestrase, anticonvulsant, antidepressant, antimicrobial, anxiolytic, and cytotoxic properties.^6^ In this context, the overview of phytochemical data on *H. vittatum* has revealed its ability to accumulate a variety of chemical metabolites, of which alkaloids represent the major class identified so far, e.g., belladine-, crinine-, galanthamine-, haemanthamine-, homolycorine-, lycorine-, narciclasine-, and tazettine-types,^6^ whereas the nonalkaloidal principles produced by this valued species have remarkably received much lesser attention.^7−9^ Therefore, as part of our research interest in different plants belonging to the family Amaryllidaceae,^4,10−12^ this work investigates the nonalkaloidal metabolites, the phenolic and flavonoidal contents, and the antioxidant potential of *H. vittatum* bulbs. Additionally, the potential ability of the characterized phytoconstituents **1–16** to interact with different target proteins of the severe acute respiratory syndrome coronavirus 2 (SARS-CoV-2), namely, the main protease (M^pro^), the papain-like protease (PLpro), and the RNA-dependent RNA polymerase (RdRp), was explored using the *in silico* molecular docking approach in order to widen the current search for naturally derived anti-Coronavirus Disease-2019 (COVID-19) agents.

## 2 Results and Discussion

### 2.1 Chemical Investigation of *H. vittatum*

**Figure 1:**
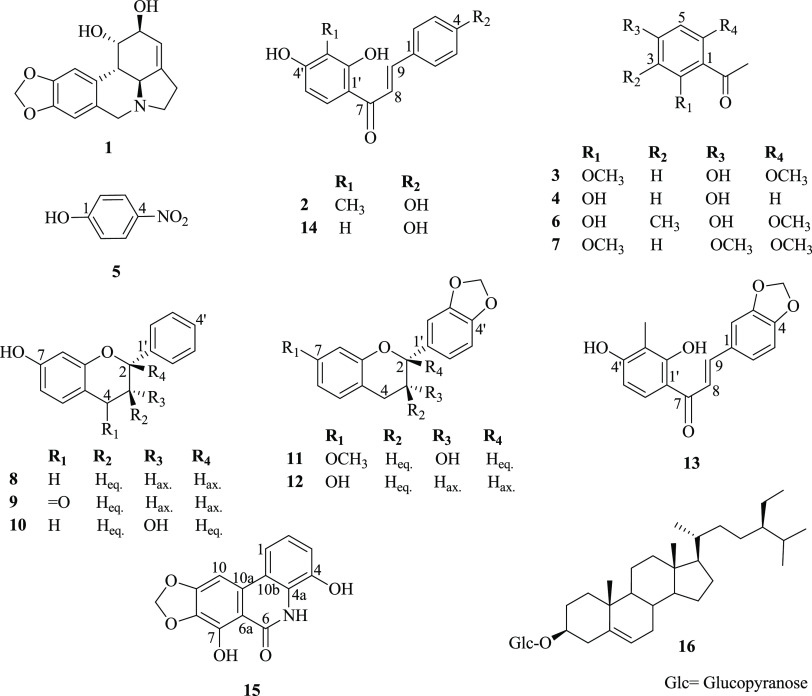
Chemical structures of the isolated compounds **1–16** from *H. vittatum* bulbs.

Repeated chromatographic fractionation and purification of the nonbasic EtOAc soluble fraction (fraction II) of the total extract of *H. vittatum* bulbs using silica gel, sephadex LH-20, polyamide, preparative RP-18 TLC, and HPLC resulted in the isolation of 16 compounds belonging to different chemical groups (Figure 1). Structures of the obtained metabolites were elucidated based on comparing their physicochemical and chromatographic properties as well as spectroscopic data (1D and 2D NMR and ESI-MS) with the literature, including 2′,4,4′-trihydroxy 3′-methylchalcone **(**3′-methyl isoliquiritigenin; **2)**,^13^ 2,6-dimethoxy-4-hydroxyacetophenone **(3)**,^14^ 2,4-dihydroxyacetophenone **(4)**,^15,16^*p*-nitrophenol **(5)**,^17^ 2,4-dihydroxy-6-methoxy-3-methylacetophenone **(6)**,^14^ 2,4,6-trimethoxyacetophenone **(7)**,^18^ 7-hydroxyflavan **(8)**,^19,20^ 7-hydroxyflavanone **(9)**,^21^ 7-hydroxyflavan-3-ol **(10)**,^20^ 7-methoxy-3′,4′-methylenedioxyflavan-3-ol **(11)**,^20^ 7-hydroxy-3′,4′-methylenedioxy flavan **(12)**,^22^ 2′,4,4′-trihydroxychalcone (isoliquiritigenin; **14)**,^13,23^ and narciprimine **(15)**,^24^ whereas both lycorine **(1)** and β-sitosterol 3-*O*-β-glucopyranoside **(16)** were identified by comparing their physical and chromatographic properties with authentic samples. In the same context, it is worthy to mention that the configuration of ring B in compounds **8**, **9**, and **12** was determined according to the coupling constants of H-2β ax. with both H-3β eq. and H-3α ax. (*J* = 2.4–3.0 and 10.0–13.5 Hz, respectively), which suggested the *pseudo*-axial and *pseudo*-equatorial orientations of H-2 and the phenyl ring B at C-2, respectively.^14,20,23^ Likewise, in compounds **10** and **11**, the small coupling constants between H-3β eq. and both H-4 ax. (*J* = 4.3 Hz) and H-4 eq. (*J* = 2–3 Hz) as well as the observed multiplicities of H-2β eq. and H-3β eq. were indicative of a 2,3-*cis*-configuration of their γ-pyran rings (ring C) and also substantiated both the *pseudo*-axial orientation of their C-3-OH group and the *pseudo*-equatorial position of H-2.^20,23,25^

**Figure 2:**
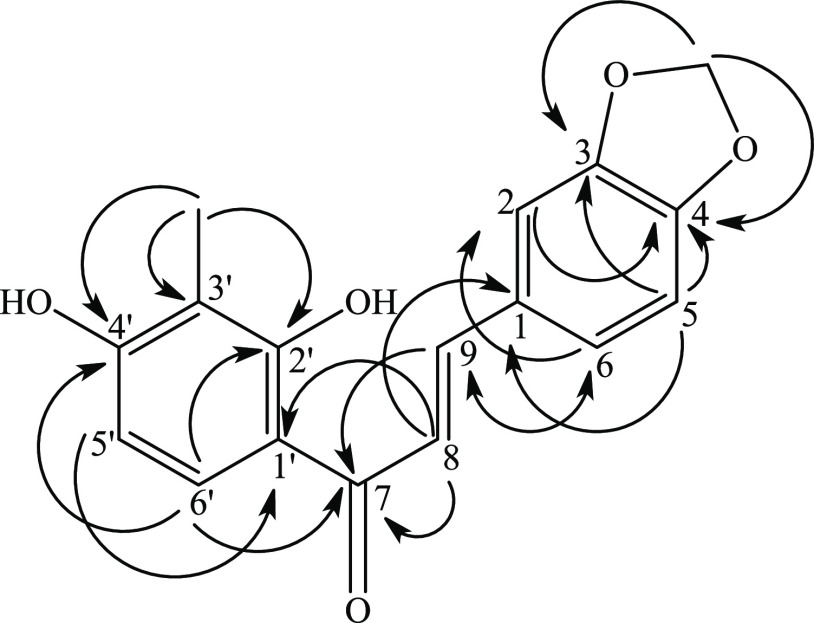
Significant HMBC correlations of compound **13**.

Apart from the abovementioned metabolites, compound **13** was obtained as a yellow amorphous powder, showing brown fluorescence under UV light and a yellow color upon exposure to ammonia vapors. It also attained a yellow color turn to orange after spraying with 10% H_2_SO_4_ reagent; such color reactions were noticeably similar to those exhibited by the chalcones **2** and **14**. Besides, preliminary investigation of the spectroscopic data of compound **13** suggested its flavonoidal nature, particularly of the chalcone-type. In this regard, the NMR data of compound **13** (Table 1) revealed two characteristic one-proton doublets at δ_H_ 7.63 (*J* = 15.0 Hz) and 7.74 (*J* = 15.0 Hz) in good agreement with the two *trans*-coupled olefinic protons H-8 and H-9, together with their corresponding carbon resonances (C-8 and C-9) at δ_C_ 120.1 and 144.8, respectively. Both carbons were simply distinguished through the observed HMBC three-bond correlations of H-9 with C-2, C-6, and C-7 in addition to those of H-8 with both C-1 and C-1′ (Figure 2). Furthermore, as a key structural feature of chalcones, the ^13^C-NMR spectrum of compound **13** displayed a quaternary carbon signal at δ_C_ 193.5, which is typical for the ketonic group (C-7) of the propenone moiety.^13^ On the other hand, the aromatic protons of the chalcone skeleton exhibited two different spin systems; the former, corresponding to ring A, was represented by two one-proton doublets at δ_H_ 6.43 (*J* = 8.9 Hz) and 7.83 (*J* = 8.9 Hz) attributable to the *ortho*-coupled protons H-5′ and H-6′, respectively,^13^ whereas those of ring B resonated as two doublets (each of one proton) at δ_H_ 6.87 (*J* = 8.0 Hz) and 7.19 (*J* = 8.0 Hz) ascribable to the *ortho*-coupled protons H-5 and H-6, respectively, along with another one-proton broad singlet at δ_H_ 7.36 for H-2, in harmony with a trisubstituted benzene ring. The NMR data of compound **13** also showed an additional three-proton singlet at δ_H_ 2.05 for an aromatic methyl group, together with its characteristic carbon resonance at δ_C_ 7.69. The attachment of this methyl group at C-3′ was confirmed by its HMBC three-bond connectivities with C-2′ and C-4′ (δ_C_ 163.4 and 165.2, respectively) as well as its two-bond correlation with C-3′ (δ_C_ 112.7). Likewise, the substitution of ring B with a methylenedioxy group was unambiguously inferred from the two-proton singlet at δ_H_ 6.02 and its characteristic carbon resonance at δ_C_ 103.1, while its position between C-3 and C-4 was established through the observed HMBC three-bond correlations of its protons with both C-3 (δ_C_ 149.7) and C-4 (δ_C_ 150.9) (Figure 2).

**Table 1:** ^1^H- and ^13^C-NMR Spectroscopic Data of Compounds **2**, **13**, and **14** (600 and 150 MHz, Methanol-*d*_4_)^a^

	2		13		14	
no.	δ_H_, (*J* in Hz)	δ_C_	δ_H_, (*J* in Hz)	δ_C_	δ_H_, (*J* in Hz)	δ_C_
**1**		128.0		131.0		127.9
**2**	7.62 d (9.0)	131.8	7.36 br.s	107.8	7.61 d (8.4)	131.8
**3**	6.83 d (9.0)	116.9		149.7	6.84 d (8.4)	116.9
**4**		161.5		150.9		161.6
**5**	6.83 d (9.0)	116.9	6.87 d (8.0)	109.5	6.84 d (8.4)	116.9
**6**	7.62 d (9.0)	131.8	7.19 d (8.0)	126.6	7.61 d (8.4)	131.8
**7**		193.8		193.5		193.6
**8**	7.60 d (16.0)	118.6	7.63 d (15.0)	120.1	7.60 d (15.6)	118.4
**9**	7.77 d (16.0)	145.4	7.74 d (15.0)	144.8	7.77 d (15.6)	145.7
**1′**		114.4		114.3		114.2
**2′**		163.8		163.4		166.4
**3′**		112.5		112.7	6.28 d (2.4)	103.8
**4′**		165.4		165.2		167.6
**5′**	6.43 d (9.0)	108.0	6.43 d (8.9)	108.3	6.41 dd (9.0, 2.4)	109.1
**6′**	7.82 d (9.0)	130.1	7.83 d (8.9)	130.3	7.96 d (9.0)	133.4
**Me-3′**	2.06 s	7.68	2.05 s	7.69		
**–OCH**_**2**_**O–**			6.02 s	103.1		

a Assignments were confirmed by 2D HSQC and HMBC experiments.

In the same way, the ^13^C-NMR data of compound **13** (Table 1) involved five methine carbons, of which those at δ_C_ 108.3 and 130.3 were typical for C-5′ and C-6′ of ring A, respectively, while those resonating at δ_C_ 107.8, 109.5, and 126.6 were attributed to C-2, C-5, and C-6 of ring B, respectively. The lower-field carbon resonances at δ_C_ 163.4, 165.2, 149.7, and 150.9 also suggested the presence of four oxygenated quaternary carbons that were assigned to C-2′, C-4′, C-3, and C-4, respectively. All of the abovementioned assignments were completely corroborated via the HSQC and HMBC experiments and were further compared with the NMR data of the related known chalcones **2** and **14** (Table 1). Finally, the ESI-MS analysis of **13** revealed a pseudomolecular ion peak [M + H]^+^ at *m*/*z* 299 in consonance with the molecular formula C_17_H_14_O_5_, suggesting that compound **13** is 2′,4′-dihydroxy-3′-methyl-3,4-methylenedioxychalcone, which to the best of our knowledge, is a new molecule.

### 2.2 Chemotaxonomic Significance of the Isolated Phytocompounds

Extensive literature review pertaining to the characterized metabolites **1–16** indicated that only lycorine **(1)** was previously isolated from *H. vittatum* bulbs,^6,26^ whereas the phenanthridone derivative, narciprimine **(15)**, which was described from a range of Amaryllidaceae plants, e.g., *Cyrtanthus*, *Galanthus*, *Lycoris*, *Narcissus*, and *Zephyranthes* species,^12^ is first obtained herein from plants of the genus *Hippeastrum*. Likewise, among different members of the Amaryllidaceae, 3′-methyl isoliquiritigenin **(2)** was only reported from *Crinum augustum* Rox. bulbs and its 3′-demethyl derivative **14** was also formerly obtained from the bulbs of *Crinum bulbispermum* L. and *Pancratium maritimum* L.;^13,14,23^ however, both chalcones **2** and **14** are reported in this work for the first time in the genus *Hippeastrum*.

In the same framework, flavans have been commonly reported among the nonalkaloidal metabolites biosynthesized by Amaryllidaceae plants, of which 7-hydroxyflavan **8** was widely isolated from several species, including the bulbs of *Narcissus pseudonarcissus* L.,^19^*Crinum americanum* L.,^27^*Crinum moorei* Hook F.,^28^*Crinum asiaticum* L.,^29^*Habranthus brachyandrus* (Baker) Sealy (syn. *Hippeastrum brachyandrum* Baker and *Zephyranthes brachyandra* Baker),^20^ and *Brunsvigia natalensis* Baker,^30^ while 7-hydroxy-3′,4′-methylenedioxy flavan **(12)** was formerly identified from both *Zephyranthes flava* Herb. bulbs and the whole plant of *Crinum biflorum* Rottb.^22,31^ The related flavonoidal molecule, 7-hydroxyflavanone **(9)**, was also obtained before from *Crinum asiaticum* L. var. *sinicum* (Roxb. ex Herb.) Baker.^32^ Interestingly, the current work represents the first report on the isolation of compounds **8**, **9**, and **12** from the genus *Hippeastrum*. Concerning flavan-3-ol derivatives, compound **11** (7-methoxy-3′,4′-methylenedioxyflavan-3-ol) was earlier obtained from the bulbs of both *Hippeastrum ananuca* Phil. and *H. brachyandrus* as well as from the whole plant of *C. biflorum*,^20,25,31^ but it is first described herein from *H. vittatum* plants. Among different amaryllids, the related procyanidin, 7-hydroxyflavan-3-ol **(10)** was only reported from *H. brachyandrus* bulbs,^20^ whereas this is the first report of its isolation from the genus *Hippeastrum*.

**Figure 3:**
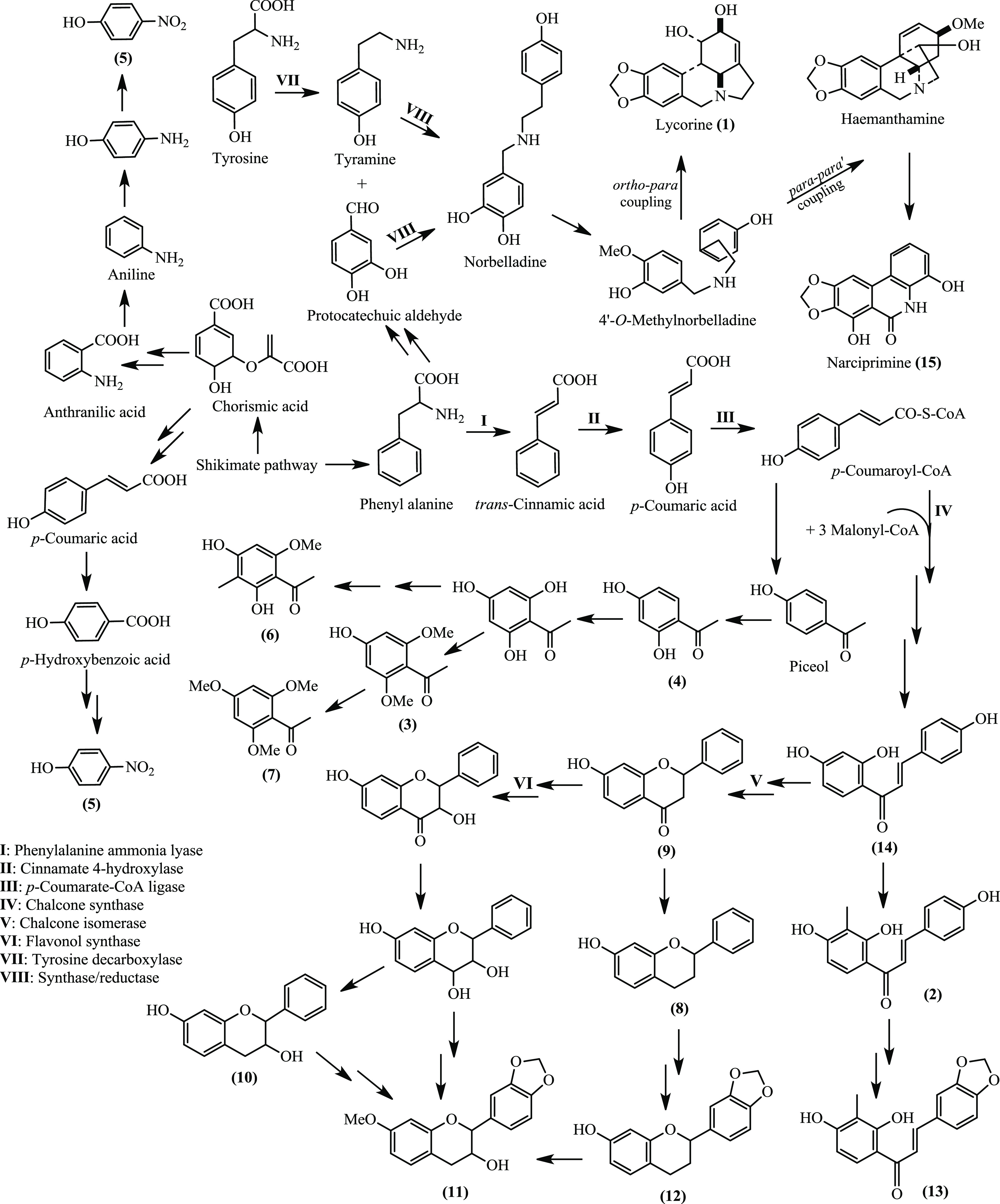
Plausible biosynthetic pathways of the isolated metabolites from *H. vittatum* bulbs.

Previous phytochemical studies on plants of the family Amaryllidaceae have also provided a group of acetophenones, among which compounds **3** and **6** were previously obtained from the bulbs of *P. maritimum*,^14^ while compound **7** was earlier purified from the flowering bulbs and the pseudostem fluid of *Pancratium biflorum* Roxb.^33^ Noteworthily, all of the aforementioned acetophenones are first identified herein in the genus *Hippeastrum*, whereas 2,4-dihydroxyacetophenone **(4)** was obtained in this study for the first time in the family Amaryllidaceae. More interestingly, the nitro-compound, *p*-nitrophenol **(5)** represents the first example of natural nitrated metabolites in the genus *Hippeastrum*. This compound was formerly described from the gastromycete, *Stephanospora caroticolor* (Berk.) Pat. (carrot truffle) and the Arctic ice bacterium, *Salegentibacter* sp. isolate T436.^34,35^ On the other hand, the common phytosterol, β-sitosterol 3-*O*-β-glucopyranoside **(16)** was reported from a number of Amaryllidaceae species;^4,10,31^ however, it was obtained in the current work for the first time from plants of the genus *Hippeastrum*. Taken together, these data reflect both the chemical homogeneity of *H. vittatum* metabolites with those biosynthesized by other amaryllids as well as the chemotaxonomic importance of different phytocompounds identified herein from *H. vittatum*. The plausible biosynthetic pathway of the isolated metabolites from *H. vittatum* is shown in Figure 3 and in comparison with those previously reported from different Amaryllidaceae plants in Schemes S1–S4.

### 2.3 Total Phenolic Content

Plant polyphenols constitute a large family of ubiquitous secondary metabolites, such as flavonoids, anthocyanins, tannins, phenolic acids, carotenoids, and tocopherols, among others, whose presence and quantitative levels have been linked to the antiradical capacities of most plants and plant-based products.^36,37^ In this respect, the Folin–Ciocalteu assay is among the most popular methods for determination of phenolics depending on the reduction of Folin–Ciocalteu reagent in the presence of phenolic compounds, resulting in the formation of molybdenum–tungsten blue, which is estimated spectrophotometrically at 760 nm. The absorption intensity of the reaction mixture is also known to increase linearly with the amount of phenolic species present in the tested sample;^38^ therefore, this test has been commonly used to measure the total phenols of a wide variety of food and medicinal plants.^37,39−41^ In view of this, the contents of total phenolic principles of the crude extract and different fractions of *H. vittatum* bulbs were assessed herein for the first time using the Folin–Ciocalteu assay. As shown in Table 2, the phenolic contents of the tested samples varied between 5.58 ± 0.19 and 23.56 ± 0.20 mg gallic acid equivalent (GAE)/g dry weight, and the highest concentration was detected in the basic EtOAc fraction III (23.56 ± 0.20 mg GAE/g), followed by the acidic EtOAc fraction II (20.70 ± 0.19 mg GAE/g) and the subfractions IV_2_ and IV_3_ of the aqueous fraction (20.45 ± 0.10 and 18.65 ± 0.18 mg GAE/g, respectively). These findings are also in line with the previous studies conducted on some Amaryllidaceae species, where the maximum amount of total phenolics was contained in their DCM and/or EtOAc extracts, including *Hippeastrum puniceum* (Lam.) Voss, *Galanthus krasnovii* A. P. Khokhr., and *Galanthus woronowii* Losink.^42−44^

**Table 2:** Results of Total Phenolic and Flavonoid Contents and Antioxidant Assays of the Total Extract and Different Fractions of *H. vittatum* Bulbs

sample	total phenolic content (mg GAE/g)	total flavonoid content (mg rutin equivalent/g)	total antioxidant capacity (mg ascorbic acid equivalent/g)	DPPH radical scavenging activity [IC_50_ (μg/mL)]^a^
total ethanol extract	10.48 ± 0.26	2.14 ± 0.20	8.47 ± 0.26	285.20 ± 0.60**
petroleum ether fraction (I)	5.61 ± 0.15	1.63 ± 0.14	3.61 ± 0.15	984.30 ± 0.61*
acidic EtOAc fraction (II)	20.70 ± 0.19	8.62 ± 0.18	8.70 ± 0.19	97.57 ± 0.37***
basic EtOAc fraction (III)	23.56 ± 0.20		9.56 ± 0.20	85.45 ± 0.38***
100% H_2_O subfraction (IV_1_)	5.58 ± 0.19	0.94 ± 0.02	4.58 ± 0.19	906.30 ± 0.70*
MeOH–H_2_O (50:50) subfraction (IV_2_)	20.45 ± 0.10	7.42 ± 0.13	22.45 ± 0.10	93.61 ± 0.48***
100% MeOH subfraction (IV_3_)	18.65 ± 0.18	6.45 ± 0.25	16.44 ± 0.26	107.50 ± 0.42***
BHT				0.96 ± 0.03

a Statistically significant differences compared with BHT [*(*P* < 0.05), **(*P* < 0.01), and ***(*P* < 0.001)].

### 2.4 Total Flavonoid Content

The flavonoid contents of the total extract and different fractions of *H. vittatum* bulbs were determined for the first time using the AlCl_3_ colorimetric assay. This method depends on the complexing ability of AlCl_3_ with flavonoids and has been widely employed for the estimation of such metabolites in several plant samples.^41,45−47^ In this respect, the estimated flavonoid contents of *H. vittatum* samples were found to range between 0.94 ± 0.02 and 8.62 ± 0.18 mg rutin equivalent/g dry weight as displayed in Table 2. Among them, the acidic EtOAc fraction II exhibited the maximum flavonoid content (8.62 ± 0.18 mg rutin equivalent/g), followed by the subfractions IV_2_ and IV_3_ of the aqueous fraction (7.42 ± 0.13 and 6.45 ± 0.25 mg rutin equivalent/g, respectively). Similar results have been also reported for the EtOAc extract of *H. puniceum* that revealed the highest amount of flavonoids among the different tested extracts of the plant.^42^

### 2.5 *In Vitro* Antioxidant Activities

The total antioxidant capacities of the total extract and fractions of *H. vittatum* bulbs were compared using the phosphomolybdenum assay that depends on the reduction of the phosphomolybdate ion in the presence of antioxidant entities, leading to the production of a green complex, which is measured spectrophotometrically at 695 nm.^48^ The obtained data (Table 2) revealed the varied antioxidant aptitudes of the studied samples of *H. vittatum* bulbs, among which both the subfractions IV_2_ (MeOH–H_2_O (50:50)) and IV_3_ (100% MeOH) demonstrated the highest antioxidant capacities (22.45 ± 0.10 and 16.44 ± 0.26 mg ascorbic acid equivalent/g). In contrast, the total ethanol extract and its derived EtOAc fractions (II and III) showed relatively mild antioxidant abilities in the range of 8.47–9.56 mg ascorbic acid equivalent/g, whereas the least phosphomolybdate reducing potential was noted for both the petroleum ether fraction I and subfraction IV_1_ (100% H_2_O) (<5.0 mg ascorbic acid equivalent/g) (Table 2).

On the other hand, the 2,2′-diphenyl-1-picrylhydrazyl (DPPH) assay was used to evaluate the free radical scavenging potential of the total extract and fractions of *H. vittatum* bulbs in comparison with butylated hydroxytoluene (BHT). The obtained results indicated that all of the tested samples exhibited dose-dependent DPPH scavenging properties, of which the basic EtOAc fraction III had the maximum inhibitory potential, with an IC_50_ value of 85.45 ± 0.38 μg/mL, followed by subfraction IV_2_, the acidic EtOAc fraction II, and subfraction IV_3_ (IC_50_ = 93.61 ± 0.48, 97.57 ± 0.37, and 107.5 ± 0.42 μg/mL, respectively), whereas the petroleum ether fraction I was the least active one. However, the observed inhibitory activities of all of the tested samples were markedly lower than BHT in terms of IC_50_ values (Table 2). Taken together, the higher antioxidant capacities and free radical scavenging potential of fractions II and III as well as of subfractions IV_2_ and IV_3_ of *H. vittatum* bulbs compared with the other fractions seem to be underpinned by their greater contents of total phenolics and/or flavonoids. In agreement with this, previous reports on a number of Amaryllidaceae plants, e.g., *G. krasnovii* and *G. woronowii*, have indicated the superior antioxidant potential of their moderately polar and polar extracts in consonance with their higher levels of phenolic metabolites.^43,44^ Noteworthily, to the best of our knowledge, this work is the first report on both the phenolic and flavonoid contents and the antioxidant properties of *H. vittatum*.

### 2.6 Molecular Docking Studies

Over the past two years, humankind has been awfully attacked by the SARS-CoV-2, or as later named COVID-19, leading to huge health, economic, and social implications.^49^ This new virus has been responsible for an estimate of 639.6 million confirmed cases and 6.6 million deaths to date.^50^ As the COVID-19 outbreak surged quickly worldwide, several protocols were adopted to manage the escalating infections, comprising the repurposing of a panel of existing drugs, e.g., antibiotic, antimalarial, and antiviral medications, or testing of possible drug combinations;^51^ however, no effective treatment has been specifically developed for COVID-19 as yet.^52^ Although the development of some vaccines against SARS-CoV-2 has raised expectations regarding the probable end of such unprecedented pandemic, the use of these vaccines has provoked concerns about some possible side effects and adverse interactions.^53,54^ Besides, the capability of the said vaccines of complete disease prevention also remains to be witnessed, which underlines the importance of identifying new therapeutic agents with considerable efficacy and safety levels.^55^ During that journey, numerous studies have been introduced to stop the replication of SARS-CoV-2, focusing on a group of viral proteins as potential therapeutic targets, e.g., helicases, polymerases, and proteases, among others.^52,56−61^ A large body of literature has therefore considered the application of virtual screening and molecular docking simulations as reasonable tools to mine for possible anti-SARS-CoV-2 candidates.^52,56−63^

**Figure 4:**
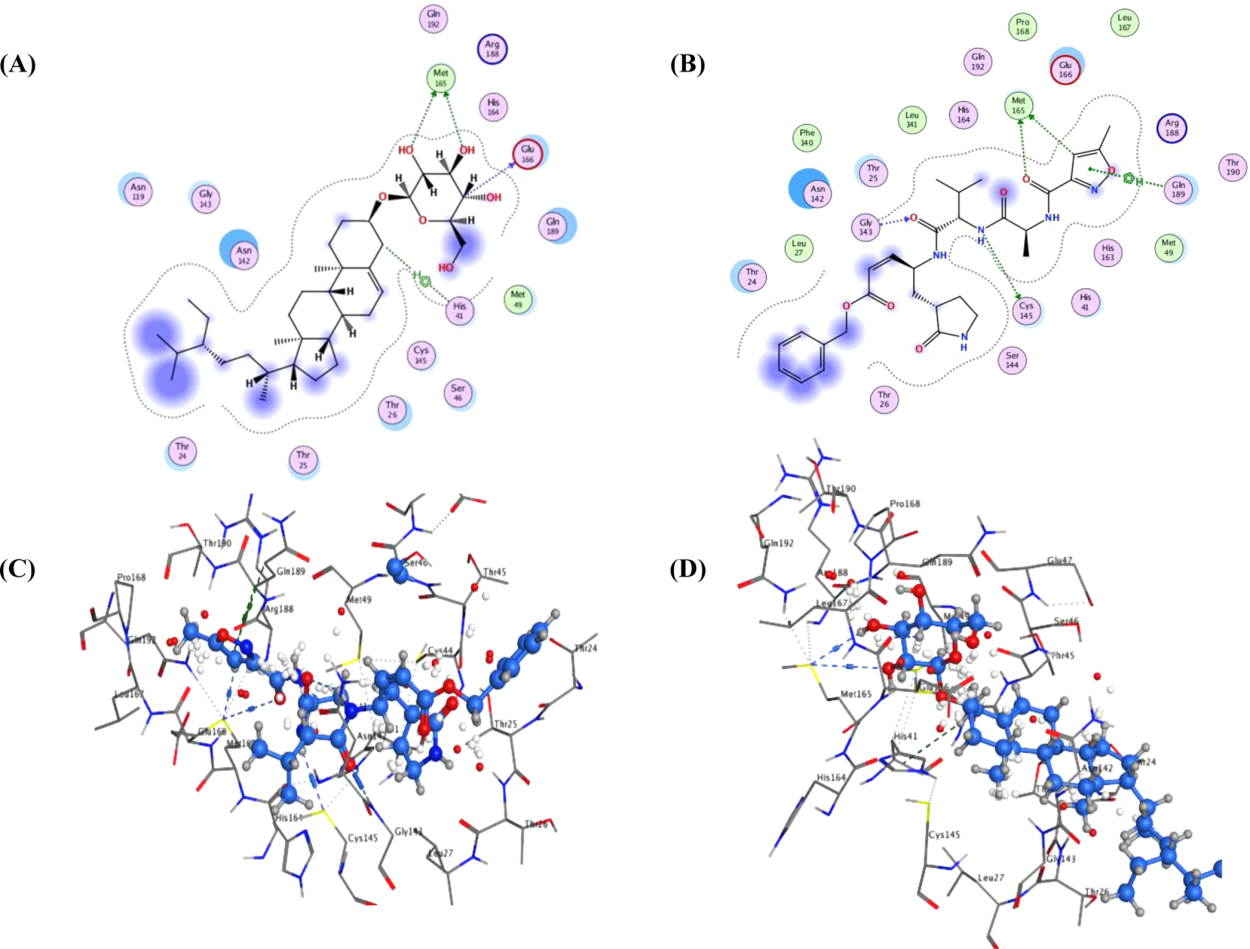
Ligand interactions of compound **16** and N3 docked into the active site of SARS-CoV-2 M^pro^ ((A, B) 2D poses of compound **16** and N3; (C, D) 3D poses of compound **16** and N3, respectively).

**Figure 5:**
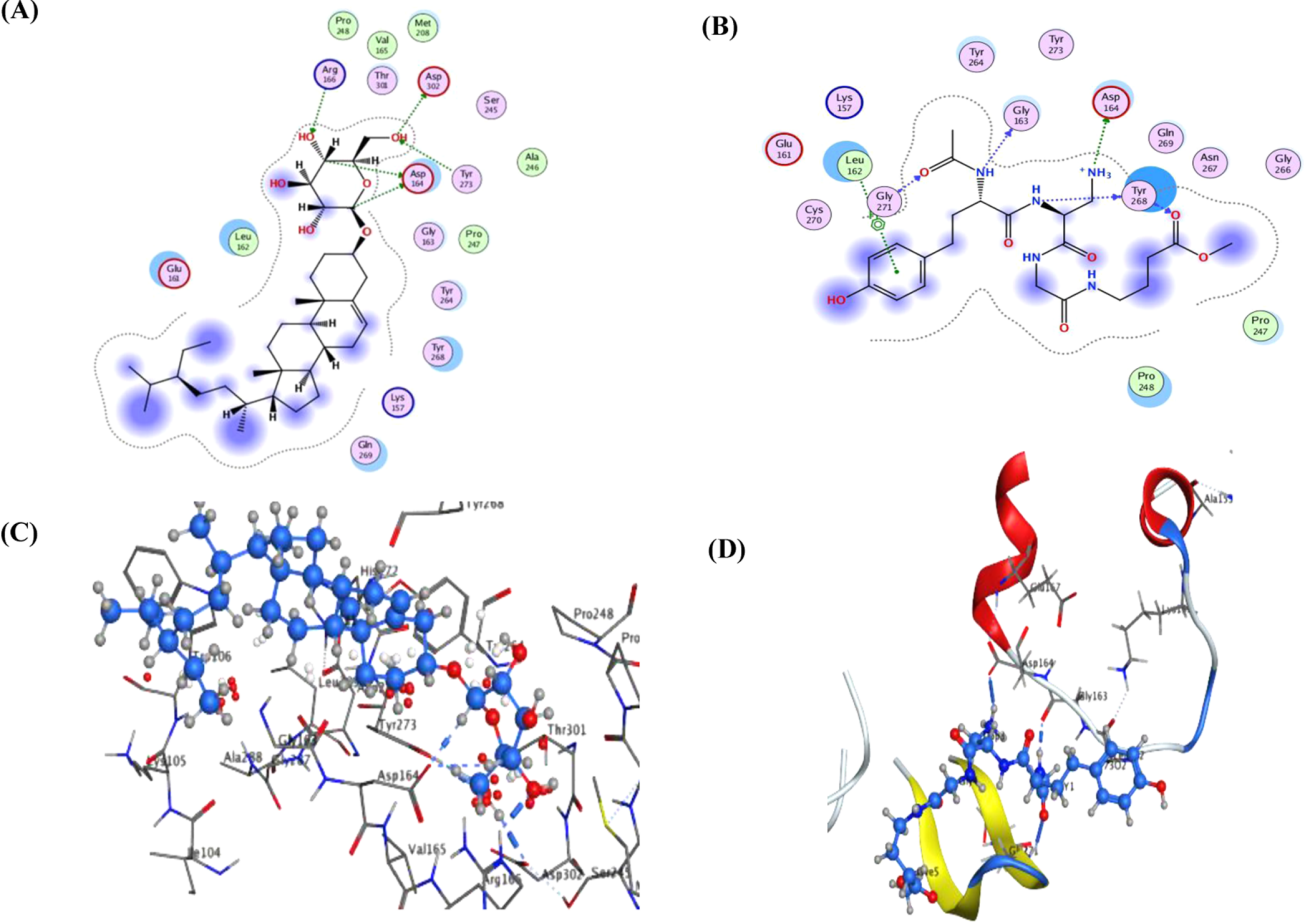
Ligand interactions of compound **16** and VIR251 docked into the active site of SARS-CoV-2 PLpro ((A, B) 2D poses of compound **16** and VIR251; (C, D) 3D poses of compound **16** and VIR251, respectively).

**Figure 6:**
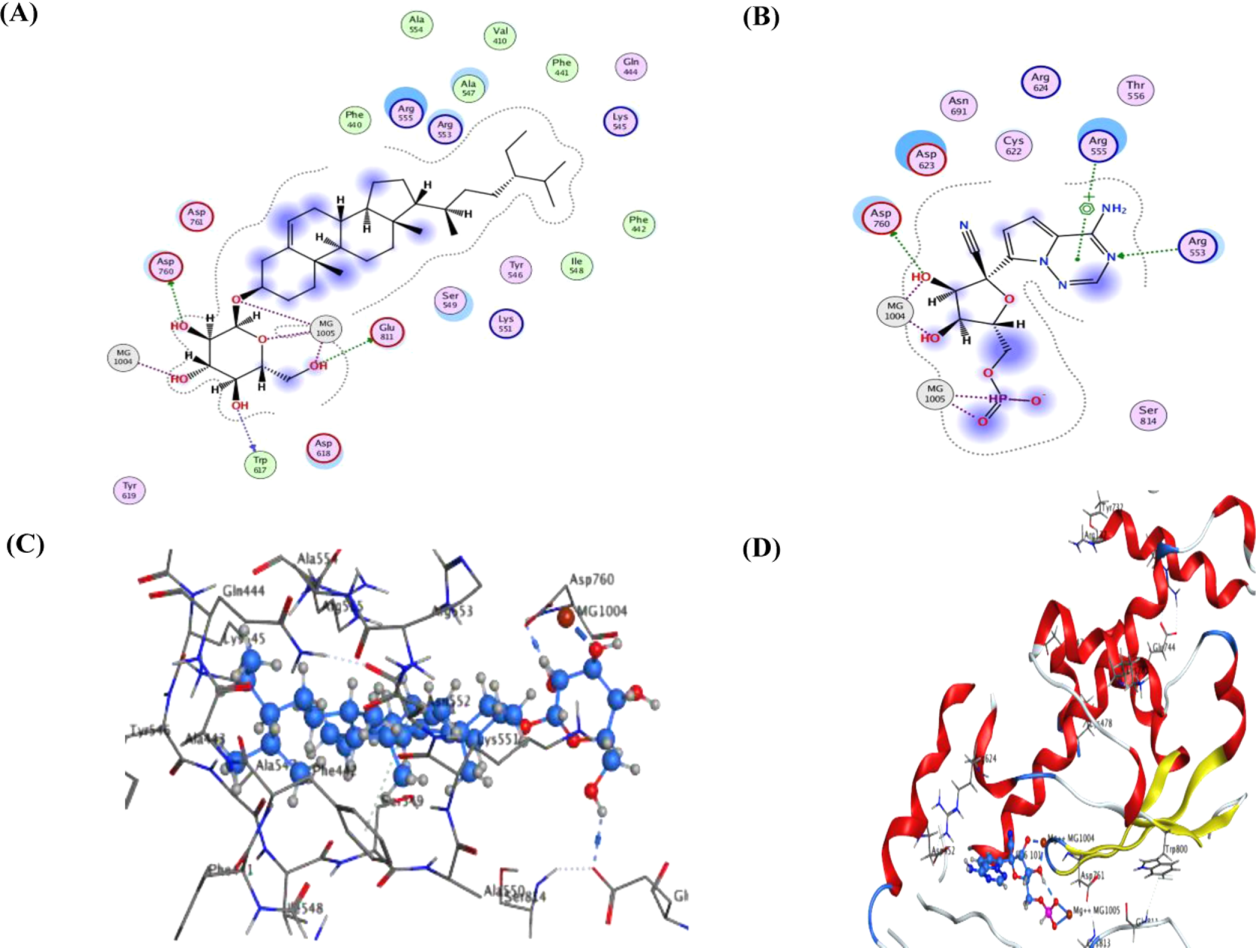
Ligand interactions of compound **16** and remedesivir docked into the active site of SARS-CoV-2 RdRp ((A, B) 2D poses of compound **16** and remedesivir; (C, D) 3D poses of compound **16** and remedesivir, respectively).

In this context, natural products have long been employed as an outstanding chemical basis for drug development, with those biosynthesized by members of the family Amaryllidaceae have increasingly become an attractive source of many drug leads.^3,11^ Prompted by this, the current docking approach focuses on testing the potential of compounds **1–16** to inhibit three significant targets of SARS-CoV-2, namely, the M^pro^ (3CL^pro^; nsp5), PLpro (nsp3), and RdRp (nsp12). Of these, the two proteases studied herein have been stated to mediate the cleavage of polyproteins into individual nonstructural proteins (nsp) as a vital step for viral life and replication.^57,64^ The obtained results were recorded as binding energy scores together with the possible ligand interactions with amino acid residues present in the catalytic sites of the aforementioned proteins (Table 3, Figures 4–6, and Tables S1–S3).

**Table 3:** Energy Scores (kcal/mol) and Types of Interactions of Compound **16** with Different Amino Acid Residues in the Binding Sites of SARS-CoV-2 M^pro^, PLpro, and RdRp in Comparison with N3, VIR251, and Remdesivir, Respectively

compound	energy score (*S*) (kcal/mol)	amino acid residues (bond type and length (Å))
***M***^***pro***^**:**		
**compound 16**	–8.28	HIS 41 (H–Pi; 4.49), MET 165 (H-bond; 3.55), MET 165 (H-bond; 3.86), GLU 166 (H-bond; 3.19)
**N3**	–8.30	GLY 143 (H-bond; 3.06), CYS 145 (H-bond; 3.79), MET 165 (H-bond; 3.74), MET 165 (H-bond; 3.48), GLN 189 (Pi–H; 3.67), GLN 189 (Pi–H; 4.08)
***PLpro*****:**		
**compound 16**	–7.59	ASP 164 (H-bond; 3.38), ASP 164 (H-bond; 3.16), ARG 166 (H-bond; 2.94), TYR 273 (H-bond; 3.15), ASP 302 (H-bond; 3.12)
**VIR251**	–7.85	LEU 162 (Pi–H; 3.95), GLY 163 (H-bond; 3.05), ASP 164 (ionic; 3.02), ASP 164 (H-bond; 3.02), TYR 268 (H-bond; 2.95), TYR 268 (H-bond; 3.14), GLY 271 (H-bond; 2.91)
***RdRp*****:**		
**compound 16**	–11.30	TRP 617 (H-bond; 2.83), ASP 760 (H-bond; 2.67), GLU 811 (H-bond; 3.16), Mg 1004 (metal; 2.17), Mg 1005 (metal; 1.98), Mg 1005 (metal; 2.05), and Mg 1005 (metal; 2.57)
**remdesivir**	–9.85	ARG 553 (H-bond; 3.04), ARG 553 (H-bond; 3.37), ARG 555 (Pi–cation; 3.48), ASP 760 (H-bond; 2.77), Mg 1004 (Ionic; 2.15), Mg 1005 (ionic; 2.04), Mg 1005 (ionic; 2.18), Mg 1005 (metal; 2.04), Mg 1005 (metal; 2.18)

Generally, the obtained data revealed the notable fitting of the tested metabolites into the binding pockets of M^pro^ and PLpro enzymes, showing moderate to promising energy scores in the ranges of −4.35 to −8.28 and −4.51 to −7.59 kcal/mol, respectively (Tables S1 and S2). Among them, β-sitosterol 3-*O*-β-glucopyranoside **(16)** showed the most stable complexes within the active sites of both enzymes (−8.28 and −7.59 kcal/mol, respectively) with comparable stability to those formed by the corresponding peptide reference agents, N3 (−8.30 kcal/mol) and VIR251 (−7.85 kcal/mol) (Table 3). The considerable binding aptitude of compound **16** to SARS-CoV-2 M^pro^ was mainly brought about by its hydrogen bonding with the key amino acids GLU 166 and MET 165 as well as its H–Pi interaction with HIS 41 (Table 3; Figure 4), while the predicted hydrogen bond interactions with ASP 164, ASP 302, ARG 166, and TYR 273 underlay its potential inhibition of SARS-CoV-2 PLpro (Table 3 and Figure 5). Some of these interactions were similar to those observed for the reference agents docked herein, including the hydrogen bonds of N3 with MET 165 and those of VIR251 with ASP 164 within the active sites of M^pro^ and PLpro, respectively. In the same vein, the rest of metabolites identified from *H. vittatum*, including alkaloids, acetophenones, and flavonoid derivatives, formed reasonably stable complexes within the substrate-binding sites of both enzymes but with lower energy scores in comparison with the tested standard inhibitors. The formation of such complexes was mostly mediated by both hydrogen bond and Pi–H interactions with a range of amino acid residues, except for compounds **2** and **14** that did not show fundamental interactions inside the active site of PLpro, despite having good free energy scores of −5.54 and −5.27 kcal/mol, respectively (Tables S1 and S2). In this regard, the capacity of natural polyphenolic metabolites, encompassing different flavonoid subtypes, to counteract the proteolytic activity of SARS-CoV-2 enzymes has been previously reported,^58,59,65−67^ a fact that could suggest the possible structural optimization of such natural molecules from *H. vittatum* in order to best appreciate their helpful role against COVID-19.

On the other hand, RdRp is an enzyme responsible for viral RNA replication in host cells, and since it has no host cell homologues, it is considered one of the most intriguing targets for anti-SARS-CoV-2 drug development.^68^ So far, a group of nucleoside analogues was researched as potential RdRp inhibitors, such as ribavirin, favipiravir, galidesivir, and redmesivir, of which the latter has acquired an emergency FDA approval for use against COVID-19 as an RdRp inhibitor.^69,70^ Remedesivir is known to compete with ATP and hampers RNA elongation via RdRp, which eventually stops viral replication.^70^ Consequently, the possible interactions of compounds **1–16** to RdRp were explored and compared with remdesivir as a reference standard. As summarized in Tables 3 and S3, the docked metabolites exhibited moderate to interesting binding potential to RdRp, with energy scores ranging from −4.69 to −11.30 kcal/mol. Compound **16** demonstrated the most pronounced binding affinity to this SARS-CoV-2 polymerase, with an energy score of −11.30 kcal/mol, which was even superior to that recorded by the antiviral drug, remdesivir (−9.85 kcal/mol) (Table 3). The binding capacity of compound **16** was attributed to the significant hydrogen bonds formed between its sugar moiety and the amino acid residues TRP 617, ASP 760, and GLU 811, along with its four metal interactions with magnesium atoms (Mg 1004 and Mg 1005) found in the active site of RdRp (Table 3 and Figure 6). Such predicted interactions of **16** were largely similar to those displayed by remedesivir within the binding site of RdRp, including the hydrogen bonding with ASP 760 and the interaction with Mg 1005 atoms, while remedesivir exhibited two additional hydrogen bond interactions with the ARG 553 and ARG 555 residues (Table 3 and Figure 6). These results also tie well with previous literature data that have supported the potential of phytosterols against SARS-CoV-2 by targeting its vital proteins.^71−73^ Such steroidal metabolites have also been suggested to contribute to the action of certain Chinese medicines used for fighting COVID-19.^73^ In this respect, the binding potential of plant steroids with a number of SARS-CoV-2 proteins, including the steroidal glycoside **16**, has been described in the literature,^58,71,74,75^ while the *in vitro* inhibitory activity of this common natural metabolite against SARS-CoV-2 has been recently reported.^76^

On the other hand, the chalcone derivatives **2**, **13**, and **14** exhibited the ability to form relatively stable complexes with RdRp, with energy scores ranging from −5.84 to −6.54 kcal/mol (Table S3). The presence of a relatively large aromatic group (ring B) in compound **13** seemed to contribute to the stability of the formed complex compared with the smaller phenyl groups observed in the related metabolites **2** and **14**. In this respect, some synthetic chalcones carrying a relatively small indole moiety have been reported to display potential interactions with SARS-CoV-2 RdRp,^77^ which supports the results observed herein. Similarly, among the docked acteophenones **3**, **4**, **6**, and **7** from *H. vittatum*, the substitution of their aromatic rings with several bulkier methoxy groups also appeared to enhance the stability of the formed complexes with the key SARS-CoV-2 proteins studied herein.

## 3 Materials and Methods

### 3.1 General Experimental Procedures

NMR experiments were recorded on Bruker DRX 600 MHz (Bruker Daltonics Inc., MA) and Bruker Avance HD III 400 MHz (Uster, Switzerland) spectrometers in DMSO-*d*_6_ and methanol-*d*_4_. ESI-MS analyses were carried out using Bruker Daltonics micro QTOF focus (Switzerland). Column chromatography (CC) was achieved on silica gel 60 (60–120 mesh; E. Merck, Darmstadt, Germany), polyamide (E-Merck, Germany), sephadex LH-20 (0.25 mm, Pharmacia), and diaion HP-20 (Sigma-Aldrich, Germany), while silica gel GF_254_ for thin layer chromatography (TLC; El-Nasr Company for Pharmaceuticals and Chemicals, Egypt) was employed for vacuum liquid chromatography (VLC). High-performance liquid chromatography (HPLC) purifications were carried out on KNAUER HPLC (smart line pump 1000, degasser, DAD) with a UV detector, using a semi-prep RP-18 column (5 μm, 10 mm × 250 mm; Waters XBridge, Germany). Solvents of HPLC grade, e.g., acetonitrile (CH_3_CN) and methanol (MeOH) (SDFCL sd Fine-Chem Limited, India), were used for HPLC work. Precoated plates of silica gel 60 GF_254_ and silica gel 60 RP-18 F_254_ (E. Merck, Darmstadt, Germany; 20 cm × 20 cm, 0.25 mm in thickness) were employed for different TLC analyses. Spots were sprayed with a 10% H_2_SO_4_ solution in ethanol, followed by heating at 110 °C for visualization. Flavonoids were also detected on TLC plates using ammonia vapors. Ascorbic acid, AlCl_3_.6H_2_O, BHT, DPPH, Folin–Ciocalteu reagent, gallic acid, Na_2_CO_3_, and rutin were purchased from Sigma-Aldrich Co., St. Louis, MO.

### 3.2 Plant Material

Bulbs of *H. vittatum* were collected at the preflowering stage from the plants cultivated in El-Orman garden, Cairo, Egypt and verified by Prof. Nasser Barakat, Botany Department, Faculty of Science, Minia University, Egypt. A voucher herbarium sample (Mn-Ph-Cog-029) was kept in Pharmacognosy Department, Faculty of Pharmacy, Minia University, Egypt.

### 3.3 Extraction and Fractionation

The air-dried, coarsely powdered bulbs (2.5 kg) of *H. vittatum* were extracted by maceration with 95% ethanol (5 L × 4). The combined macerate was filtered, and the solvent was evaporated under vacuum to a syrupy consistency (220.0 g). The crude extract of bulbs was then suspended in distilled water (500 mL) and defatted with light petroleum ether (60–80 °C) in a separating funnel. The combined petroleum ether washings were concentrated under vacuum to provide fraction I (34.5 g). The aqueous layer was then acidified with tartaric acid (10%) and extracted with successive portions of ethyl acetate (EtOAc) that were combined and concentrated under vacuum to yield fraction II (35.0 g). Afterward, the mother liquor was completely basified with Na_2_CO_3_, leading to the precipitation of a creamy powder that was thoroughly washed with small volumes of MeOH to afford compound **1** as white needles (80.0 mg; m.p. 261–262 °C). The mother liquor was then re-extracted with successive portions of EtOAc, and the combined EtOAc washings were concentrated under vacuum to give fraction III (33.5 g). Finally, the remaining aqueous layer was concentrated under vacuum to a semisolid viscous residue (fraction IV; 110.0 g).

### 3.4 Isolation of Compounds **1–16**

The acidic EtOAc fraction II (35.0 g) was subjected to VLC fractionation on silica gel (4.5 cm × 45 cm; 300 g) using dichloromethane (DCM)–MeOH gradient mixtures (0, 2, 5, 10, 20, and 100%) to afford six subfractions [II_1_ (0.7 g), II_2_ (10.0 g), II_3_ (10.0 g), II_4_ (5.0 g), II_5_ (5.5 g), and II_6_ (3.5 g)]. Of these, II_2_ (10.0 g) was fractionated on a silica gel column (6 cm × 98 cm; 500 g) into five subfractions (II_2_–F–1: II_2_–F–5) applying gradient elution with petroleum ether–EtOAc. II_2_–F–2 (1.95 g) was further subjected to sephadex LH-20 CC (2.5 × 98 cm; 100 g) using DCM–MeOH (1:1) to give six subfractions (II_2_–F–2–a: II_2_–F–2–f), of which II_2_–F–2–c (0.7 g) was rechromatographed on a sephadex LH-20 column (2 cm × 80 cm; 85.0 g) using MeOH to finally provide three subfractions. Compound **2** (7.0 mg) was precipitated from II_2_–F–2–c1 (170.0 mg) and repurified by washing with successive volumes of MeOH. II_2_–F–2–c2 (285.0 mg) was also purified on a polyamide column (2 cm × 70 cm; 80.0 g) using MeOH as the mobile phase to give **3** (9.0 mg) and **4** (8.0 mg), whereas HPLC purification of II_2_–F–2–c3 using 5% CH_3_CN for 5 min, then a linear gradient to 100% CH_3_CN within 55 min, and finally with an isocratic condition of CH_3_CN for 5 min at 2 mL/min afforded **5** (5.0 mg; *R*_t_ = 25.9 min) and **6** (15.0 mg; *R*_t_ = 29.9 min).

Likewise, II_2_–F–2–d (0.7 g) was fractionated by CC on sephadex LH-20 (2 cm × 80 cm; 85.0 g) using MeOH to yield four subfractions (II_2_–F–2–d1:II_2_–F–2–d4), of which II_2_–F–2–d2 (280.0 mg) was further chromatographed on a polyamide column (2 cm × 70 cm, 80.0 g) using MeOH, providing three subfractions (II_2_–F–2–d2–1: II_2_–F–2–d2–3). Compound **7** (10.0 mg) was obtained from II_2_–F–2–d2–1 (85.0 mg) by preparative RP-18 TLC using MeOH–H_2_O (60:40) as the mobile phase. II_2_–F–2–d2–2 (125.0 mg) was also subjected to HPLC purification using 5% CH_3_CN for 5 min, followed by a linear gradient to 100% CH_3_CN within 50 min and finally with a further isocratic condition of CH_3_CN for 5 min at 2 mL/min to give **8** (8.0 mg; *R*_t_ = 26.0 min), **9** (6.0 mg; *R*_t_ = 26.3 min), **10** (4.0 mg; *R*_t_ = 26.7 min), and **11** (6.0 mg; *R*_t_ = 26.8 min). Similarly, subfraction II_2_–F–2–d4 (190.0 mg) was purified on a silica gel column (0.5 cm × 50 cm; 12.0 g) employing gradient elution with petroleum ether–DCM to furnish **12** (12.0 mg) and **13** (7.0 mg), while **14** (8.0 mg) and **15** (30.0 mg) were obtained from II_2_–F–2–e (0.2 g) and II_2_–F–2–f (0.2 g), respectively, via precipitation. II_2_–F–4 (2.95 g) also yielded a white precipitate that was purified by washing with MeOH to afford **16** (30.0 mg).

On the other hand, fraction IV (110.0 g) of the total bulbs’ extract was deprived of its mucilaginous content by dissolving it in a minimum amount of distilled water, and the solution was poured into MeOH to help the precipitation of mucilage, which was washed with several portions of MeOH, yielding a yellowish white powder (70.0 g). The water-soluble part of IV was then concentrated, and the resulting residue (40.0 g) was finally fractionated on diaion HP-20 (95 cm × 3.5 cm) using H_2_O, MeOH–H_2_O (50:50), and MeOH to obtain three subfractions [IV_1_ (29.0 g), IV_2_ (8.0 g), and IV_3_ (2.0 g), respectively]. A little part of each of these fractions was used for total phenolic and flavonoid determinations and for *in vitro* antioxidant testing, whereas their major amounts were kept for future phytochemical investigation.

### 3.5 Estimation of Total Phenolic Content

The total phenolic content was estimated by the Folin–Ciocalteu method as described in the literature.^38^ Analysis was performed by adding 3.5 mL of deionized water, 50 μL of the sample extract/fraction, 50 μL of Folin–Ciocalteu reagent (2N), and 300 μL of 10% Na_2_CO_3_ solution. The reaction was left for 30 min, and the absorbance was obtained at 760 nm. The blank consisted of all reagents except for the tested sample. A standard curve was made using gallic acid, and total phenolic contents were provided as mg of GAE per gram of the dry extract/fraction weight. All determinations were carried out in triplicate.

### 3.6 Estimation of Total Flavonoid Content

The total flavonoid content was determined according to the published literature,^78^ where 0.3 mL of the total extract/fractions, 3.4 mL of 30% MeOH, 0.15 mL of NaNO_2_ (0.5 M), and 0.15 mL of AlCl_3_.6H_2_O (0.3 M) were mixed, followed by the addition of 1 mL of NaOH solution (1 M) after 5 min. The solution was thoroughly mixed, and the absorbance was obtained against the blank at 506 nm. The standard curve was prepared using a standard solution of rutin at different concentrations following the same procedure. Total flavonoid contents were calculated as milligrams of rutin equivalent per gram of the dry extract/fraction weight. Analyses were presented in triplicate.

### 3.7 Antioxidant Assays

#### 3.7.1 Phosphomolybdenum Assay

The total antioxidant capacity was studied by the phosphomolybdenum method.^48^ An aliquot of 0.1 mL of the sample solution was mixed with 1 mL of the reagent solution (sodium phosphate (28 mM), ammonium molybdate (4 mM), and sulfuric acid (0.6 M)). The tubes were capped and incubated for 90 min at 95 °C using a water bath. After cooling, the absorbance of the reacted mixture was obtained at 695 nm against a blank, which composed of 1 mL of the reagent solution and the suitable volume of the solvent and was kept under the same conditions. A standard curve was prepared using ascorbic acid, and the antioxidant activity was presented relative to that of ascorbic acid (mg ascorbic acid equivalent/g dry weight). All experiments were carried out in triplicate.

#### 3.7.2 DPPH Radical Scavenging Assay

The free radical scavenging potential was measured using the DPPH assay.^79,80^ Briefly, 200 μL of either the total extract or fractions at various concentrations were added to 2 mL of DPPH solution (0.1 mM). The mixture was shaken and incubated in the dark for 15 min at the normal room temperature. Methanol was used instead of the tested samples as a control, while BHT was employed as a reference compound. The absorbance was obtained at 517 nm, and the ability of samples to scavenge the DPPH radical was determined using the following equation
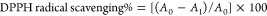
where *A*_0_ represents the absorbance of the control reaction mixture and A_1_ is that of the sample. The extract concentration providing 50% inhibition (IC_50_) was then calculated by plotting the percentage of DPPH scavenging against sample concentration. The assay was carried out in triplicate.

### 3.8 Statistical Analysis

Data were expressed as mean ± S.D. One-way analysis of variance (ANOVA), followed by Dunnett’s test was applied. Graph Pad Prism 7 was used for statistical calculations (Graph Pad Software, San Diego, California). Results were considered significant at *p* values less than 0.05, 0.01, and 0.001.

### 3.9 Molecular Docking Studies

Compounds **1–16** were docked into the active sites of SARS-CoV-2 M^pro^ [PDB: 7BUY as the crystal structure of M^pro^ in complex with carmofur; 1.6 Å],^81^ PLpro [PDB: 6WX4 as the crystal structure of SARS-CoV-2 PLpro in complex with the peptide inhibitor, VIR251; 1.66 Å],^82^ and RdRp [PDB: 7BV2 as the nsp12–nsp7–nsp8 complex bound to the template–primer RNA as well as triphosphate form of remdesivir; 2.5 Å]^83^ using Molecular Operating Environment (MOE). PDB files were obtained from the Protein Data Bank at https://www.rcsb.org. Proteins were prepared, hydrogen atoms were added, atoms were corrected for any errors, potentials were fixed, and charges were parameterized. Molecular docking was then done after energy minimization of the 3D structures of the tested compounds to a RMSD gradient of 0.1 kcal/mol and 0.1 Å. The protein was set as rigid, α triangle as the placement methodology, and London dG as the scoring methodology. Ligands were redocked into the prepared proteins to validate the method used. Docking was done as original ligands were identified as the binding site; the obtained poses were evaluated using energy scores and ligand interactions with the key amino acid residues in the active site of each protein. Poses showing the greatest energy scores and ligand–enzyme interactions were chosen and recorded.

## 4 Conclusions

The present study provided the first detailed investigation of the nonbasic metabolites of *H. vittatum* bulbs, particularly with respect to their phenolic constituents, which led to the isolation and identification of a group of compounds of different structural types for the first time in the genus *Hippeastrum*. Beyond the rich pool of Amaryllidaceae alkaloids reported in *H. vittatum*, the current findings indicated the capacity of this species to produce a diversity of nonalkaloidal principles that showed appreciable chemical homogeneity with those biosynthesized by other members of the subfamily Amaryllidoideae, thus valorizing the potential chemotaxonomic role of these metabolites. Additionally, results of the current research could also provide a basis for further investigation of different extracts of *H. vittatum* plants, especially concerning the antioxidant and anti-SARS-CoV-2 potential of their phytoconstituents, taking into consideration the possible structural modification of such natural scaffolds with the intention to develop targeted anti-COVID-19 agents.

## Supporting Information Available

Spectroscopic data, plausible biosynthetic pathways, and molecular docking results of compounds **1–16** from *H. vittatum* (PDF)
