# Supplementary material for: Studies on the Nonalkaloidal Secondary Metabolites of Hippeastrum vittatum (L’Her.) Herb. Bulbs
Source: ACS Omega. 2023 Jul 23;8(30):26749–61. doi: 10.1021/acsomega.2c07886 (PMC10398848; doi:10.1021/acsomega.2c07886)
Supplement: Supplementary file 1 — ao2c07886_si_001.pdf [file ao2c07886_si_001.pdf]

Supporting Information

Studies on the nonalkaloidal secondary metabolites of *Hippeastrum vittatum* (L'Her.) Herb. bulbs

Marwa Fathy Khalifa[a]†, John Refaat Fahim[*a]†, Ahmed E. Allam[b], Mai E. Shoman[c], Amr El Zawily[*d,e]

Mohamed Salah Kamel[a], Kuniyoshi Shimizu[f], Eman Zekry Attia[a]†

[a] Department of Pharmacognosy, Faculty of Pharmacy, Minia University, 61519 Minia, Egypt.

[b] Department of Pharmacognosy, Faculty of Pharmacy, Al-Azhar University, 71524 Assiut, Egypt.

[c] Department of Medicinal Chemistry, Faculty of Pharmacy, Minia University, 61519 Minia, Egypt.

[d] Department of Plant and Microbiology, Faculty of Science, Damanhour University, 22511 Damanhour, Egypt.

[e] Department of Biology, University of Iowa, Iowa City, IA 52242-1324, USA.

[f] Department of Agro-Environmental Sciences, Graduate School of Bioresource and Bioenvironmental Sciences, Kyushu University, 744 Motooka, Nishi-ku, 819-0395 Fukuoka, Japan.

†Those authors have equally contributed to this work.

[*]Corresponding authors:

Amr El Zawily: amr-elzawily@uiowa.edu

John Refaat Fahim: john.michael@mu.edu.eg

I) Spectral data of the isolated compounds 1–16 from *H. vittatum* bulbs

Lycorine (1)

White needles (m.p. 261–262 °C). It was identified by comparison of its physical and chromatographic properties with an authentic sample of lycorine. This compound was previously isolated from *H. vittatum* bulbs.[1,2]

2',4,4'-Trihydroxy-3'-methylchalcone (3'-methyl isoliquiritigenin) (2)

Yellow amorphous powder; ESI-MS: *m/z* 271 $[M+H]^+$; ^{1}H-NMR (600 MHz, CD_3OD): 7.82 (1H, d, *J*= 9.0 Hz, H-6'), 7.77 (1H, d, *J*= 16.0 Hz, H-9), 7.62 (2H, d, *J*= 9.0 Hz, H-2 and H-6), 7.60 (1H, d, *J*= 16.0 Hz, H-8), 6.83 (2H, d, *J*= 9.0 Hz, H-3 and H-5), 6.43 (1H, d, *J*= 9.0 Hz, H-5'), 2.06 (3H, s, CH_3-3'); ^{13}C-NMR (150 MHz, CD_3OD): 193.8 (C-7), 165.4 (C-4'), 163.8 (C-2'), 161.5 (C-4), 116.9 (C-3 and C-5), 145.4 (C-9), 131.8 (C-2 and C-6), 130.1 (C-6'), 128.0 (C-1), 118.6 (C-8), 114.4 (C-1'), 112.5 (C-3'), 108.0 (C-5'), 7.68 (CH_3-3'). The spectral data were consistent with those reported in the literature by Abd El-Hafiz et al. (1990).[3] This is the first report for its isolation from plants of the genus *Hippeastrum*.

2,6-Dimethoxy-4-hydroxyacetophenone (3)

White amorphous powder; ESI-MS: *m/z* 197 $[M+H]^+$; ^{1}H-NMR (600 MHz, CD_3OD): 6.09 (2H, s, H-3 and H-5), 3.74 (6H, s, OCH_3-2 and OCH_3-6), 2.39 (3H, s, CO-CH_3); ^{13}C-NMR (150 MHz, CD_3OD): 204.6 ($\underline{C}$O-CH_3), 162.5 (C-4), 160.3 (C-2 and C-6), 113.0 (C-1), 92.9 (C-3 and C-5), 56.2 (OCH_3-2 and OCH_3-6), 32.6 (CO-$\underline{C}H_3$). The spectral data were in accordance with those reported in the literature by Youssef et al. (1998).[4] This is the first report for its isolation from plants of the genus *Hippeastrum*.

2,4-Dihydroxyacetophenone (4)

Brown solid; ESI-MS: *m/z* 153 $[M+H]^+$;^{1}H-NMR (400 MHz, CD_3OD): 7.72 (1H, d, *J*= 8.8 Hz, H-6), 6.36 (1H, dd, *J*= 2.4, 8.8 Hz, H-5), 6.26 (1H, d, *J*= 2.4 Hz, H-3), 2.54 (3H, s, CO-CH_3); ^{13}C-NMR (100 MHz, CD_3OD): 202.6 ($\underline{C}$O-CH_3)165.4 (C-2), 162.7 (C-4), 132.9 (C-6), 114.6 (C-1), 108.0 (C-5), 103.7 (C-3), 26.4 (CO-$\underline{C}H_3$). The spectral data were in agreement with those reported in the literature by Li et al. (1992) and Sun et al. (2009).[5,6] This is the first report for its isolation in the family Amaryllidaceae.

p-Nitrophenol (5)

Yellow solid; ESI-MS: *m/z* 138 $[M-H]^-$;^{1}H-NMR (400 MHz, CD_3OD): 8.06 (2H, d, *J*= 9.2 Hz, H-3 and H-5), 6.72 (2H, d, *J*= 9.2 Hz, H-2 and H-6); ^{13}C-NMR (100 MHz, CD_3OD): 164.4 (C-1), 140.3 (C-4), 126.5 (C-3 and C-5), 116.7 (C-2 and C-6). The spectral data were in harmony with those reported in the

literature by Abraham and Mobli (2007).[7] This is the first report for its isolation in the family Amaryllidaceae.

2,4-Dihydroxy-6-methoxy-3-methylacetophenone (6)

White amorphous powder; ESI-MS: *m/z* 195 $[M-H]^-$; ^{1}H-NMR (400 MHz, CD_3OD): 6.01 (1H, s, H-5), 3.86 (3H, s, OCH_3-6), 2.57 (3H, s, CO-CH_3), 1.95 s (C$\underline{H}_3$-3); ^{13}C-NMR (100 MHz, CD_3OD): 203.3 ($\underline{C}$O-CH_3), 164.2 (C-2), 162.8 (C-4), 160.9 (C-6), 102.8 (C-1), 102.5 (C-3), 91.4 (C-5), 56.4 (OCH_3-6), 32.4 s (CO-$\underline{C}H_3$); 7.30 (CH_3-3). The spectral data were consistent with those reported in the literature by Youssef et al. (1998).[4] This is the first report for its isolation from plants of the genus *Hippeastrum*.

2,4,6-Trimethoxyacetophenone (7)

Brown solid; ESI-MS: *m/z* 209 $[M-H]^-$; ^{1}H-NMR (400 MHz, CD_3OD): 6.24 (2H, s, H-3 and H-5), 3.84 (3H, s, OCH_3-4), 3.80 (6H, s, OCH_3-2 and OCH_3-6), 2.42 (3H, s, CO-CH_3); ^{13}C-NMR (100 MHz, CD_3OD): 203.6 ($\underline{C}$O-CH_3), 162.4 (C-4), 158.5 (C-2 and C-6), 113.8 (C-1), 90.8 (C-3 and C-5), 56.5 (OCH_3-4), 56.3 (OCH_3-2 and OCH_3-6), 32.5 (CO-$\underline{C}H_3$). The spectral data were in accordance with those reported in the literature by Chiaradia et al. (2008).[8] This is the first report for its isolation from plants of the genus *Hippeastrum*.

7-Hydroxyflavan (8)

Yellow amorphous powder; ESI-MS: *m/z* 227 $[M+H]^+$; ^{1}H-NMR (400 MHz, CD_3OD): 7.41–7.43 (2H, m, H-3' and H-5') 7.35–7.38 (2H, m, H-2' and H-6'), 7.28 (1H, m, H-4'), 6.87 (1H, d, *J*= 8.4 Hz, H-5), 6.33 (1H, dd, *J*= 2.4, 8.4 Hz, H-6), 6.30 (1H, d, *J*= 2.4 Hz, H-8), 5.02 (1H, dd, *J*= 2.4, 10.0 Hz, H-2 ax.), 2.85 (1H, m, H-4b), 2.65 (1H, m, H-4a), 2.15 (1H, m, H-3 ax.), 1.95 (1H, m, H-3 eq.); ^{13}C-NMR (100 MHz, CD_3OD): 156.2 (C-7), 155.5 (C-9), 142.0 (C-1'), 129.5 (C-5), 127.9 (C-3' and C-5'), 127.2 (C-4'), 125.5 (C-2' and C-6'), 112.8 (C-10), 107.7 (C-6), 102.6 (C-8), 77.5 (C-2), 30.1 (C-4), 23.8 (C-3). The spectral data were in harmony with those reported in the literature by Coxon et al. (1980) and Jitsuno et al. (2009).[9,10] This is the first report for its isolation from plants of the genus *Hippeastrum*.

7-Hydroxyflavanone (9)

Greyish white powder; ESI-MS: *m/z* 241 $[M+H]^+$; ^{1}H-NMR (400 MHz, CD_3OD): 7.74 (1H, d, *J*= 8.7 Hz, H-5), 7.51 (2H, d, *J*= 7.4 Hz, H-2' and H-6'), 7.43 (2H, dd, *J*= 7.1, 7.4 Hz, H-3' and H-5'), 7.37 (1H, d, overlapped, H-4'), 6.51 (1H, dd, *J*= 2.0, 8.7 Hz, H-6), 6.39 (1H, d, *J*= 2.0 Hz, H-8), 5.53 (1H, dd, *J*= 3.0, 13.5 Hz, H-2 ax.), 3.01 (1H, dd, *J*= 13.5, 17.0 Hz, H-3 ax.), 2.76 (1H, dd, *J*= 3.0, 17.0 Hz, H-3 eq.); ^{13}C-NMR (100 MHz, CD_3OD): 192.2 (C-4), 165.2 (C-7), 163.5 (C-9), 136.5 (C-1'), 128.4 (C-5), 128.3 (C-3' and C-5'), 128.1 (C-4'), 125.9 (C-2' and C-6'), 114.8 (C-10), 109.7 (C-6), 102.5 (C-8), 79.6 (C-2),

43.7 (C-3). The spectral data were in agreement with those reported in the literature by De Olivera et al. (1972).[11] This is the first report for its isolation in the family Amaryllidaceae.

7-Hydroxyflavan-3-ol (10)

Yellow solid; ESI-MS: *m/z* 243 $[M+H]^+$; ^{1}H-NMR (400 MHz, CD_3OD): 7.50 (2H, d, *J*= 7.2 Hz, H-2' and H-6'), 7.38 (2H, dd, *J*= 7.2, 8.0 Hz, H-3' and H-5'), 7.29 (1H, d, overlapped, H-4'), 6.90 (1H, d, *J*= 8.0 Hz, H-5), 6.40 (1H, dd, *J*= 2.0, 8.0 Hz, H-6), 6.38 (1H, d, *J*= 2.0 Hz, H-8), 5.05 (1H, br. s, H-2 eq.), 4.25 (1H, m, H-3 eq.), 3.16 (1H, dd, *J*= 2.8, 16.2 Hz, H-4 eq.), 2.74 (1H, dd, *J*= 4.3, 16.2 Hz, H-4 ax.); ^{13}C-NMR (100 MHz, CD_3OD): 156.3 (C-7), 155.0 (C-9), 139.2 (C-1'), 130.2 (C-5), 127.6 (C-3' and C-5'), 127.0 (C-4'), 126.4 (C-2' and C-6'), 110.3 (C-10), 108.3 (C-6), 102.6 (C-8), 78.7 (C-2), 66.2 (C-3), 32.7 (C-4). The spectral data were in consonance with those reported in the literature by Jitsuno et al. (2009).[10] This is the first report for its isolation from plants of the genus *Hippeastrum*.

7-Methoxy-3',4'-methylenedioxyflavan-3-ol (11)

Yellowish white amorphous powder; ESI-MS: *m/z* 301 $[M+H]^+$; ^{1}H-NMR (400 MHz, CD_3OD): 7.06 (1H, d, *J*= 1.6 Hz, H-2'), 6.95 (1H, dd, overlapped, H-6'), 6.81 (1H, d, *J*= 8.0 Hz, H-5'), 6.95 (1H, d, overlapped, H-5), 6.51 (1H, dd, *J*= 2.8, 7.2 Hz, H-6), 6.47 (1H, d, *J*= 2.8 Hz, H-8), 5.95-5.96 (2H, d, *J*= 1.2 Hz, $-OCH_2O-$), 4.98 (1H, br. s, H-2 eq.), 4.19 (1H, m, H-3 eq.), 3.76 (3H, s, OCH_3-7), 3.16 (1H, dd, *J*= 3.1, 16.5 Hz, H-4 eq.), 2.75 (1H, dd, *J*= 4.3, 16.5 Hz, H-4 ax.); ^{13}C-NMR (100 MHz, CD_3OD): 159.2 (C-7), 155.1 (C-9), 147.5 (C-3'), 147.0 (C-4'), 133.1 (C-1'), 130.2 (C-5), 119.7 (C-6'), 111.4 (C-10), 107.3 (C-5'), 107.2 (C-6), 106.1 (C-2'), 101.0 (C-8), 100.8 ($-OCH_2O-$), 78.6 (C-2), 66.2 (C-3), 54.3 (OCH_3-7), 32.6 (C-4). The spectral data were in accordance with those reported in the literature by Jitsuno et al. (2009).[10] This is the first report for its isolation from *Hippeastrum vittatum*.

7-Hydroxy-3',4'-methylenedioxyflavan (12)

Yellow amorphous powder; ESI-MS: *m/z* 269 $[M-H]^-$; ^{1}H-NMR (400 MHz, CD_3OD): 6.89 (1H, d, *J*= 6.0 Hz, H-5), 6.87 (1H, d, overlapped, H-6'), 6.86 (1H, d, overlapped, H-2'), 6.79 (1H, d, *J*= 7.9 Hz, H-5'), 6.33 (1H, dd, *J*= 2.4, 8.2 Hz, H-6), 6.28 (1H, d, *J*= 2.4 Hz, H-8), 5.93-5.94 (2H, d, *J*= 1.2 Hz, $-OCH_2O-$), 4.90 (1H, dd, *J*= 2.4, 9.6 Hz, H-2 ax.), 2.81 (1H, m, H-4b), 2.63 (1H, m, H-4a), 2.10 (1H, m, H-3 ax.), 1.95 (1H, m, H-3 eq.); ^{13}C-NMR (100 MHz, CD_3OD): 156.2 (C-7), 155.6 (C-9), 147.7 (C-3'), 147.1 (C-4'), 136.0 (C-1'), 129.5 (C-5), 119.1 (C-6'), 112.8 (C-10), 107.8 (C-5'), 107.5 (C-6), 106.1 (C-2'), 102.6 (C-8), 100.9 ($-OCH_2O-$), 77.5 (C-2), 23.9 (C-3), 30.1 (C-4), 23.9 (C-3). The spectral data were consistent with those reported in the literature by Ghosal et al. (1985).[12] This is the first report for its isolation from plants of the genus *Hippeastrum*.

2′,4′-Dihydroxy-3′-methyl-3,4-methylenedioxy chalcone (13)

Yellow amorphous powder; ESI-MS: *m/z* 299 $[M+H]^+$; ^{1}H-NMR (600 MHz, CD_3OD): 7.83 (1H, d, *J*= 8.9 Hz, H-6'), 7.74 (1H, d, *J*= 15.0 Hz, H-9), 7.63 (1H, d, *J*= 16.0 Hz, H-8), 7.36 (1H, br. s, H-2), 7.19 (1H, d, *J*= 8.0 Hz, H-6), 6.87 (1H, d, *J*= 8.0 Hz, H-5), 6.43 (1H, d, *J*= 8.9 Hz, H-5') 6.02 (2H, s, $-OCH_2O-$), 2.05 (3H, s, CH_3-3'); ^{13}C-NMR (150 MHz, CD_3OD): 193.5 (C-7), 165.2 (C-4'), 163.4 (C-2'), 150.9 (C-4), 149.7 (C-3), 144.8 (C-9), 130.3 (C-6'), 131.0 (C-1), 126.6 (C-6), 120.1 (C-8), 114.3 (C-1'), 112.7 (C-3'), 109.5 (C-5), 108.3 (C-5'), 107.8 (C-2), 103.1 ($-OCH_2O-$), 7.69 (CH_3-3'). This metabolite was identified herein as a new compound.

2′,4,4′-Trihydroxychalcone (isoliquiritigenin) (14)

Yellow amorphous powder; ESI-MS: *m/z* 257 $[M+H]^+$; ^{1}H-NMR (600 MHz, CD_3OD): 7.96 (1H, d, *J*= 9.0 Hz, H-6'), 7.77 (1H, d, *J*= 15.6 Hz, H-9), 7.61 (2H, d, *J*= 8.4 Hz, H-2 and H-6), 7.60 (1H, d, *J*= 15.6 Hz, H-8), 6.84 (2H, d, *J*= 8.4 Hz, H-3 and H-5), 6.41 (1H, dd, *J*= 2.4, 9.0 Hz, H-5'), 6.28 (1H, d, *J*= 2.4 Hz, H-3'); ^{13}C-NMR (150 MHz, CD_3OD): 193.6 (C-7), 167.6 (C-4'), 166.4 (C-2'), 161.6 (C-4), 145.7 (C-9), 133.4 (C-6'), 131.8 (C-2 and C-6), 127.9 (C-1), 118.4 (C-8), 116.9 (C-3 and C-5), 114.2 (C-1'), 109.1 (C-5'), 103.8 (C-3'). The spectral data were in consonance with those reported in the literature by Abd El-Hafiz et al. (1990) and Ramadan et al. (2000).[3,13] This is the first report for its isolation from plants of the genus *Hippeastrum*.

Narciprimine (15)

Brown amorphous powder; ESI-MS: *m/z* 272 $[M+H]^+$; ^{1}H-NMR (600 MHz, DMSO-d_6): 13.8 (1H, br. s, Ar-OH), 7.71 (1H, d, *J*= 8.4 Hz, H-1), 7.53 (1H, s, H.10), 7.09 (1H, dd, *J*= 7.8, 8.4 Hz, H-2), 6.93 (1H, d, *J*= 7.8 Hz, H-3), 6.17 (2H, s, $-OCH_2O-$); ^{13}C-NMR (150 MHz, DMSO-d_6): 164.9 (C-6), 153.4 (C-9), 144.9 (C-4), 144.3 (C-7), 132.2 (C-8), 131.8 (C-10a), 124.0 (C-4a), 123.0 (C-2), 119.3 (C-10b), 113.6 (C-1), 113.3 (C-3), 107.0 (C-6a), 102.1 ($-OCH_2O-$), 93.5 (C-10). The spectral data were in accordance with those reported in the literature by Nair et al. (2011).[14] This is the first report for its isolation from plants of the genus *Hippeastrum*.

β-Sitosterol 3-O-β-glucopyranoside (16)

White amorphous powder; It was identified by comparison of its physical and chromatographic properties with an authentic sample. This is the first report for its isolation from plants of the genus *Hippeastrum*.

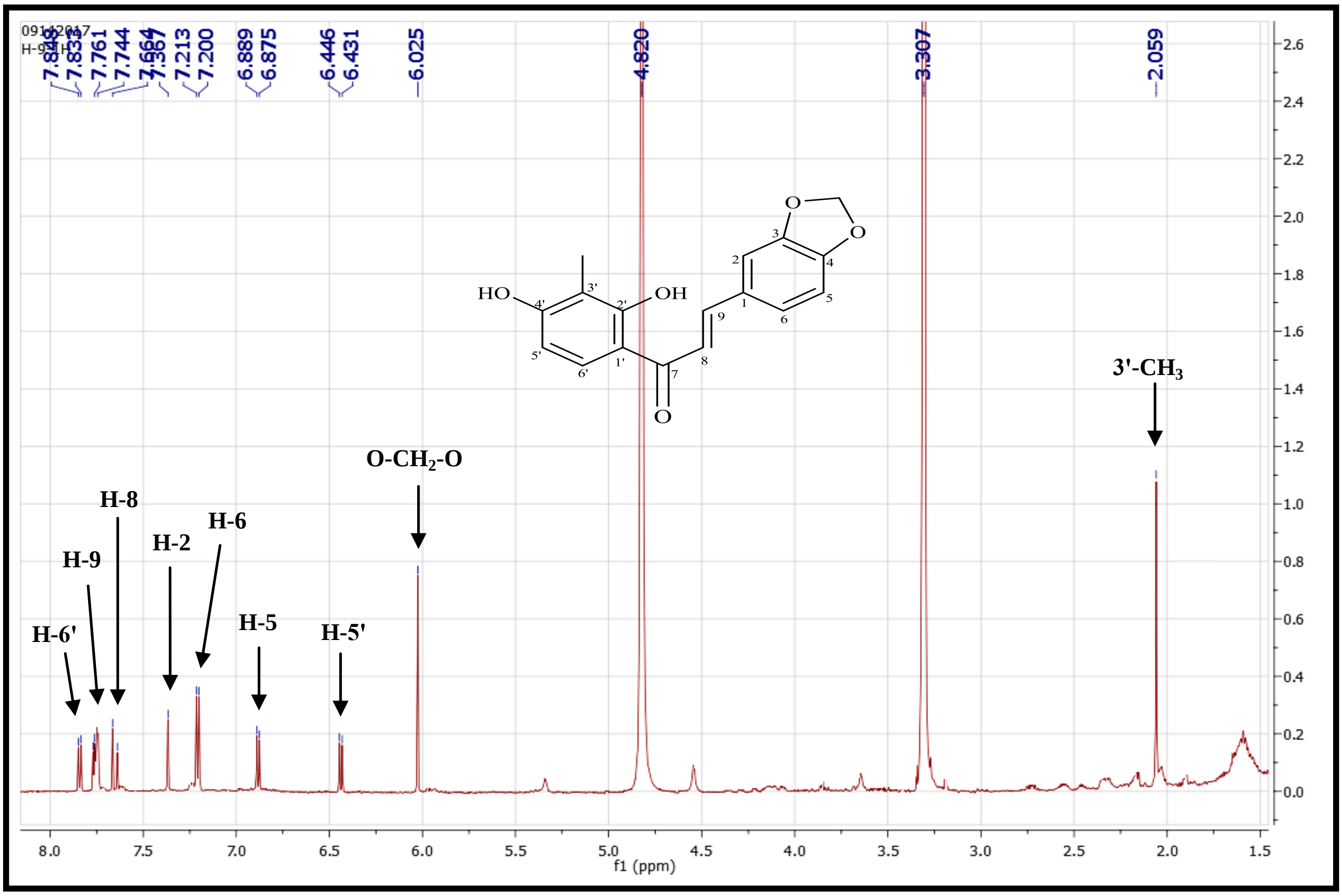

Figure S1 (a)

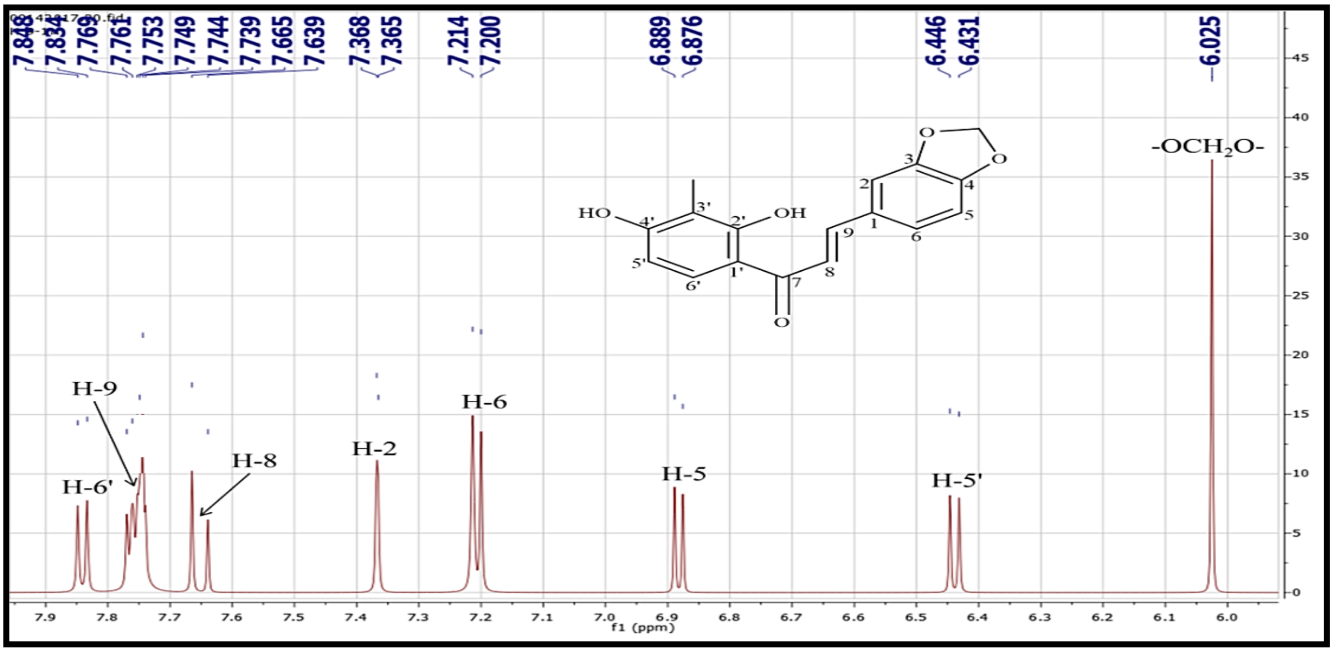

Figure S1 (b)

Figure S1 (a & b). ^{1}H-NMR spectra of compound **13** (600 MHz, MeOD).

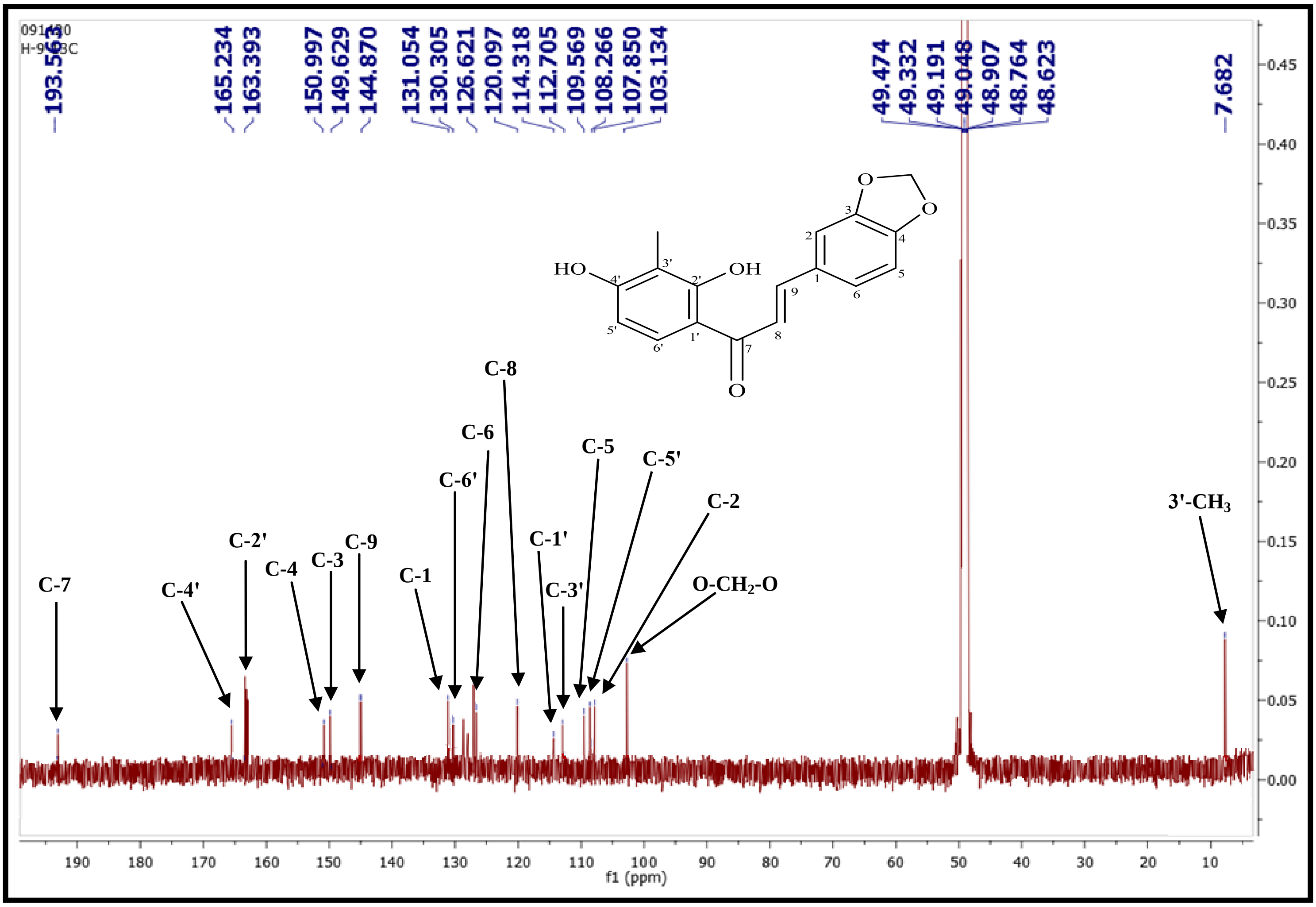

Figure S2. ^{13}C-NMR spectrum of compound **13** (150 MHz, MeOD).

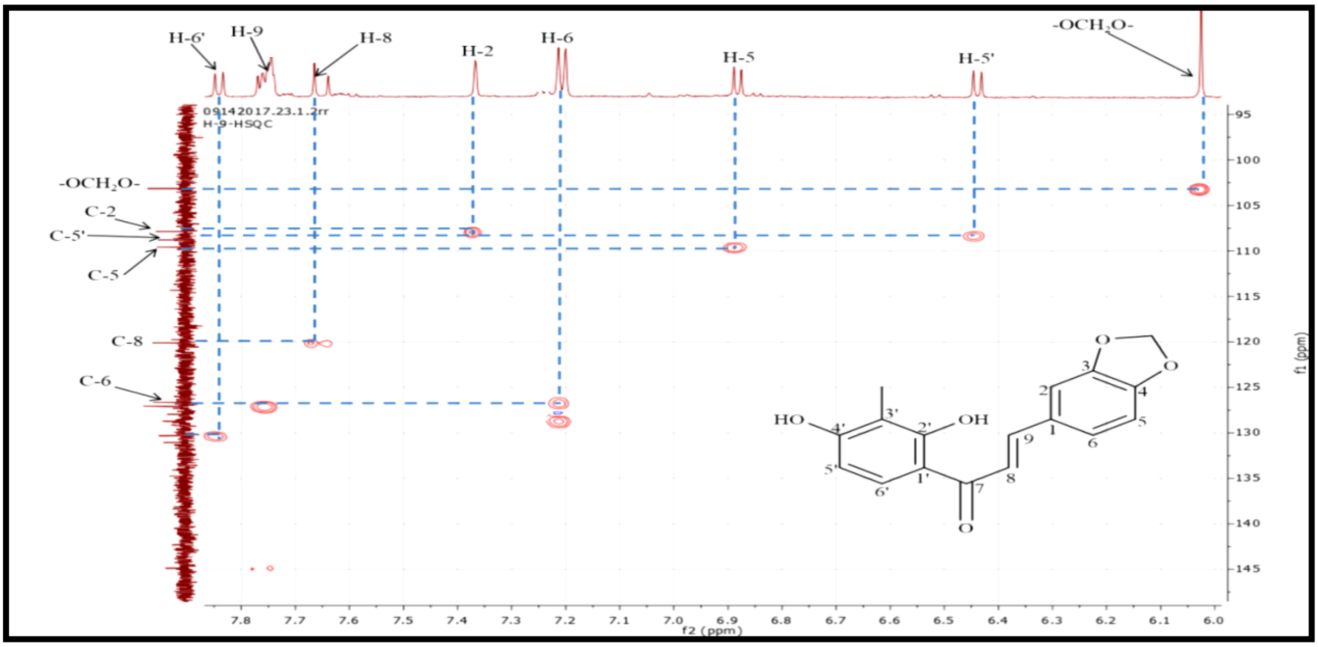

Figure S3. HSQC spectrum of compound **13**.

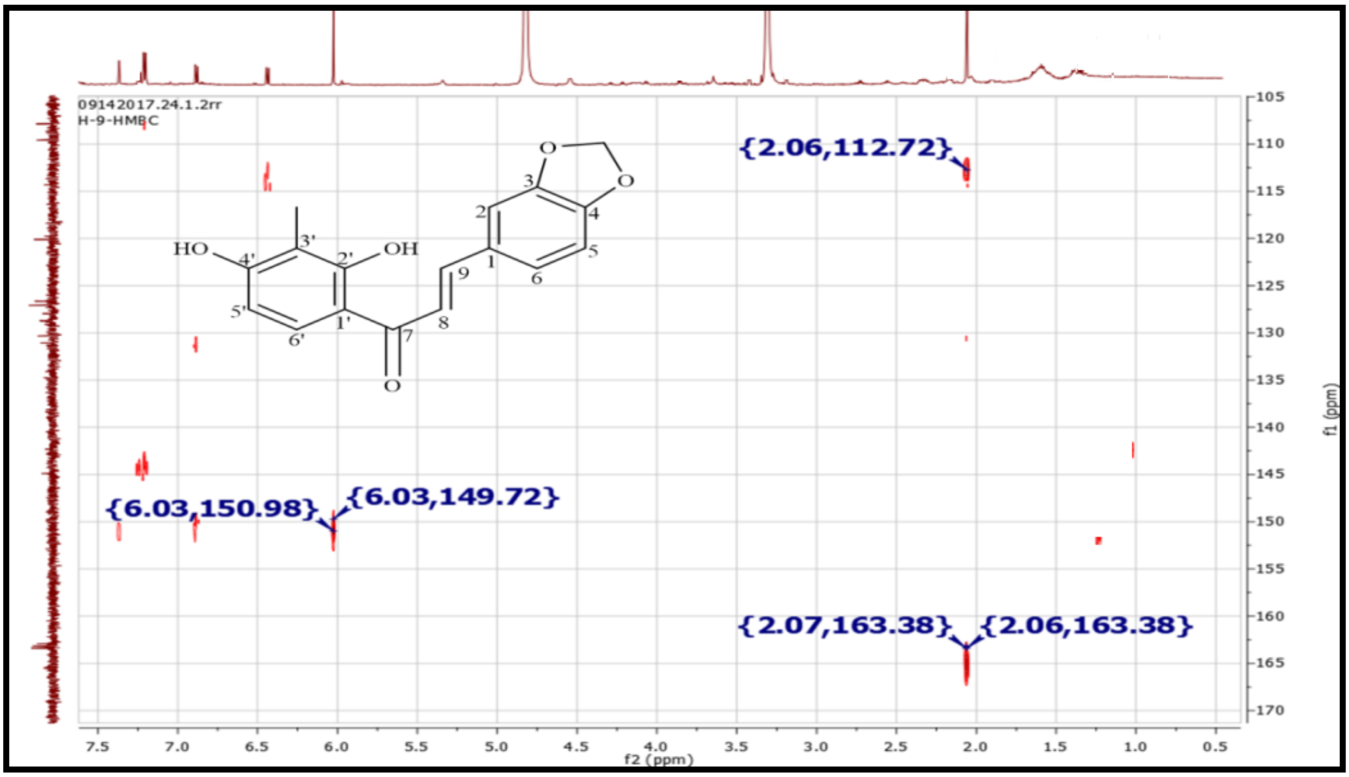

Figure S4 (a)

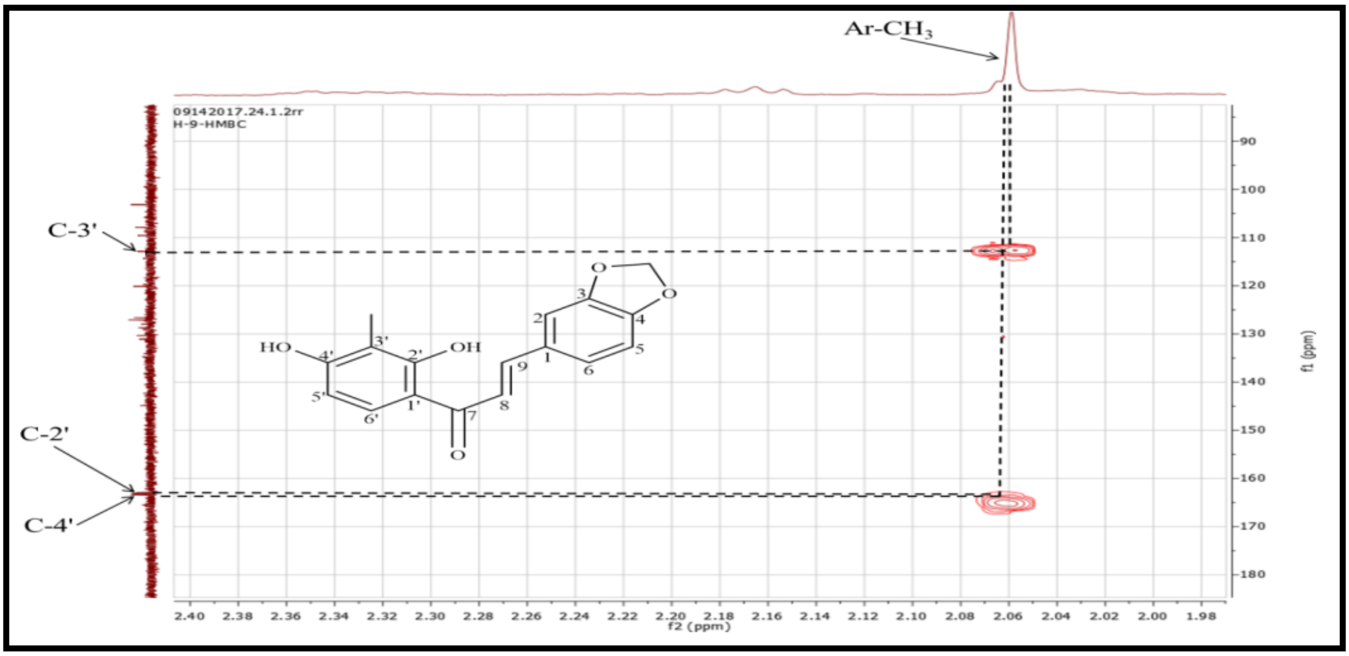

Figure S4 (b)

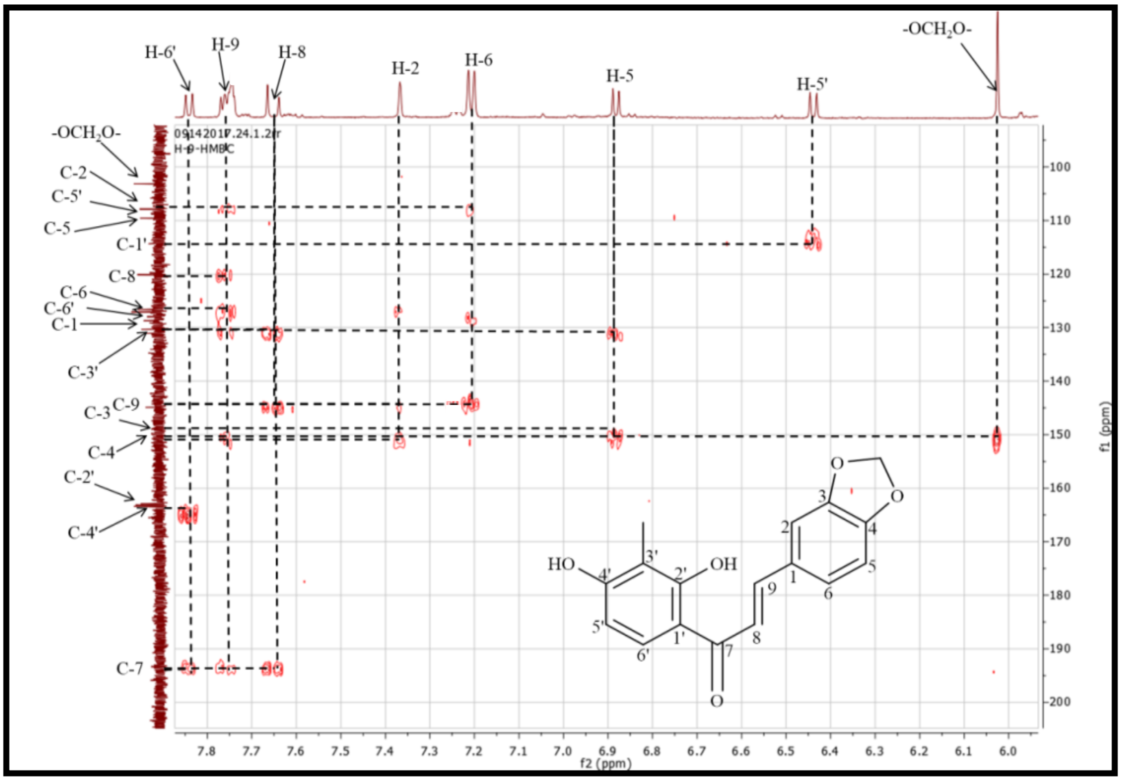

Figure S4 (c)

Figure S4 (a, b & c). HMBC spectra of compound **13**.

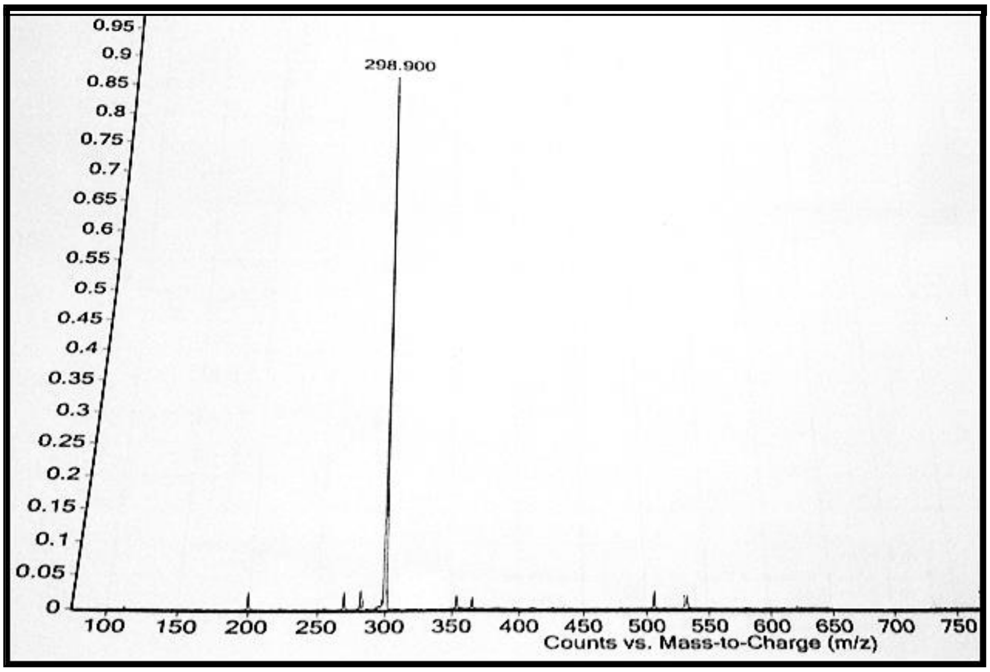

Figure S5. ESI-MS spectrum of compound **13**.

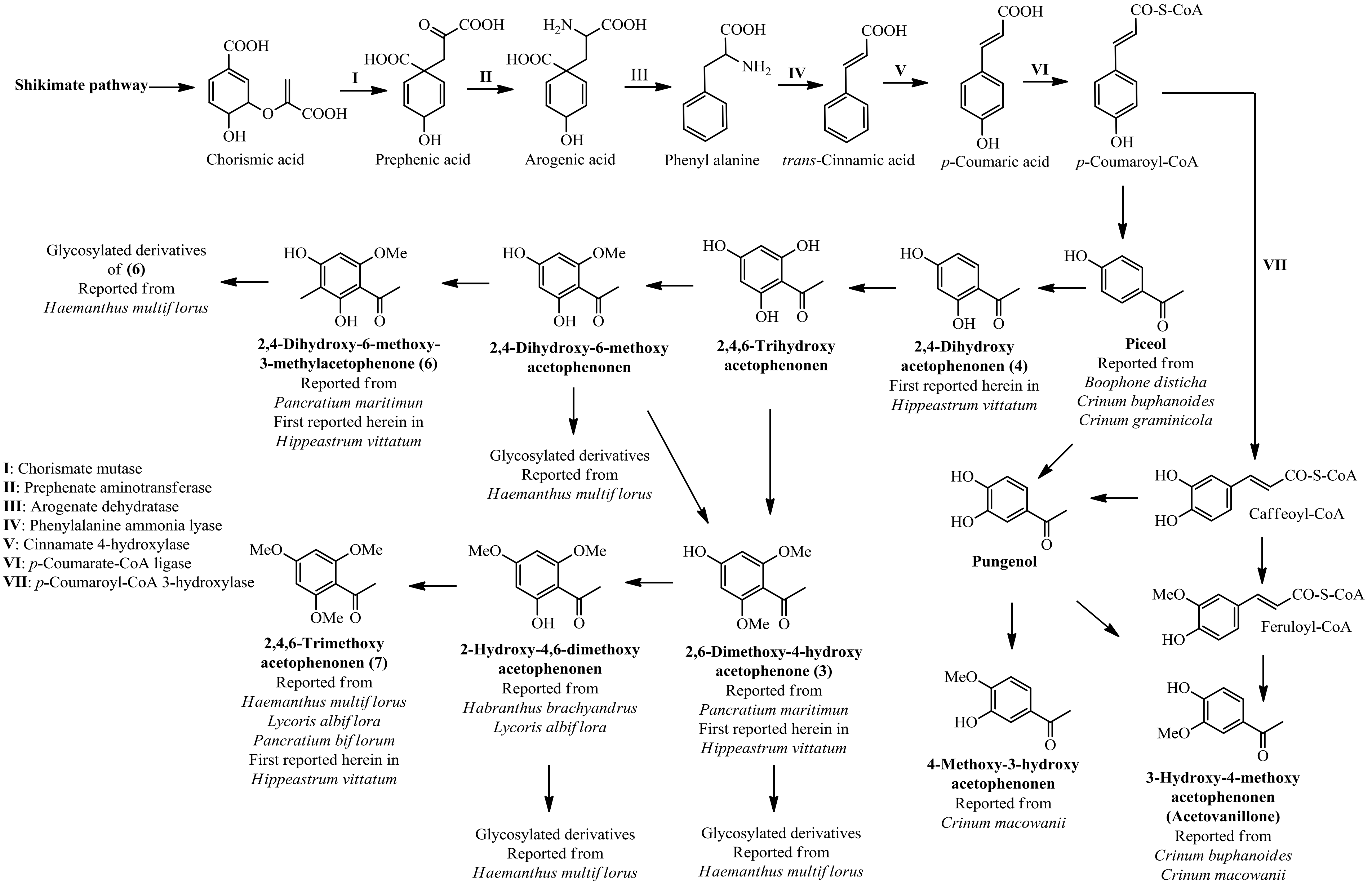

Scheme S1. Plausible biosynthetic pathway of acetophenones from *H. vittatum* in comparison with those reported from other Amaryllidaceae plants.

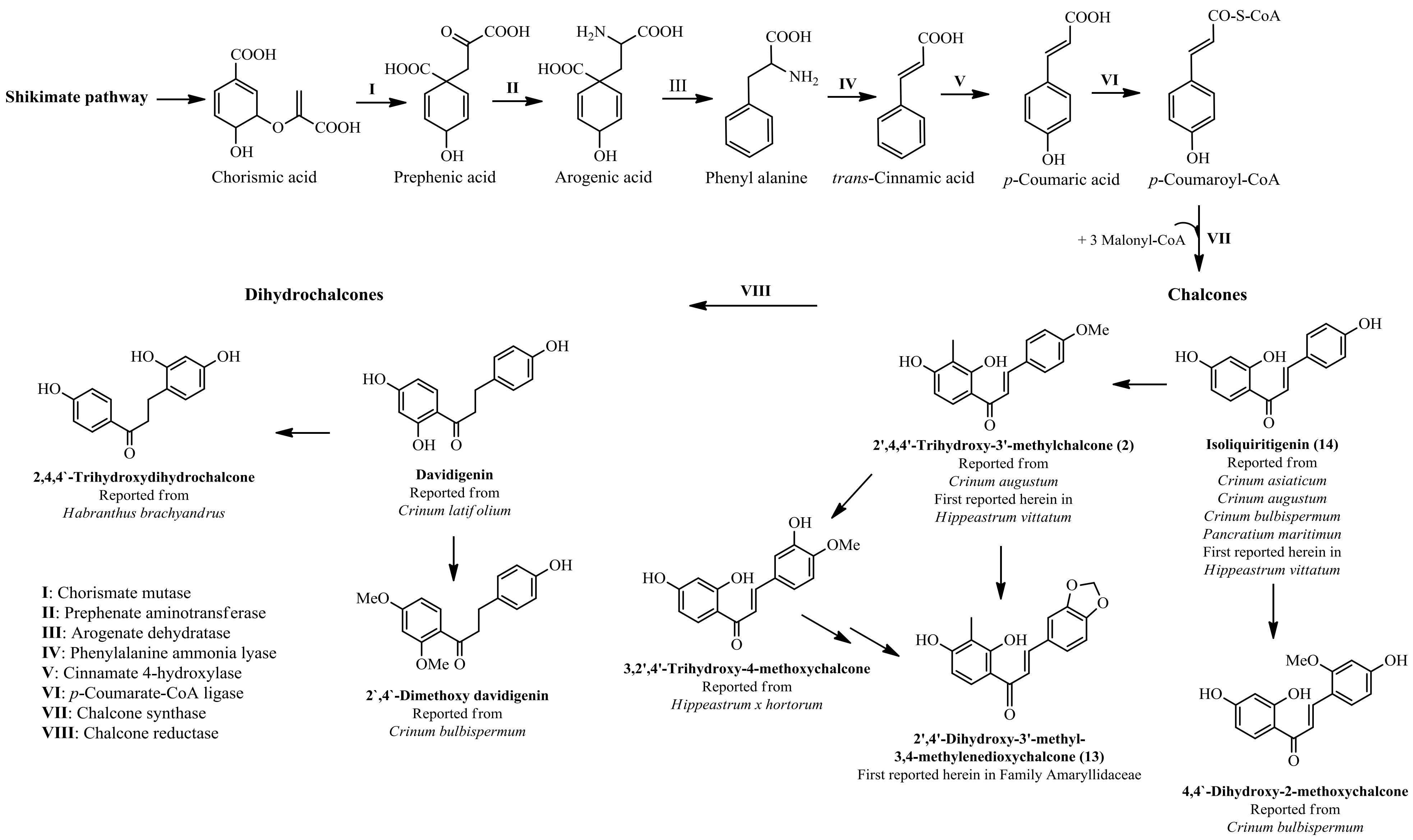

Scheme S2. Plausible biosynthetic pathway of chalcones and dihydrochalcones from *H. vittatum* in comparison with those reported from other Amaryllidaceae plants.

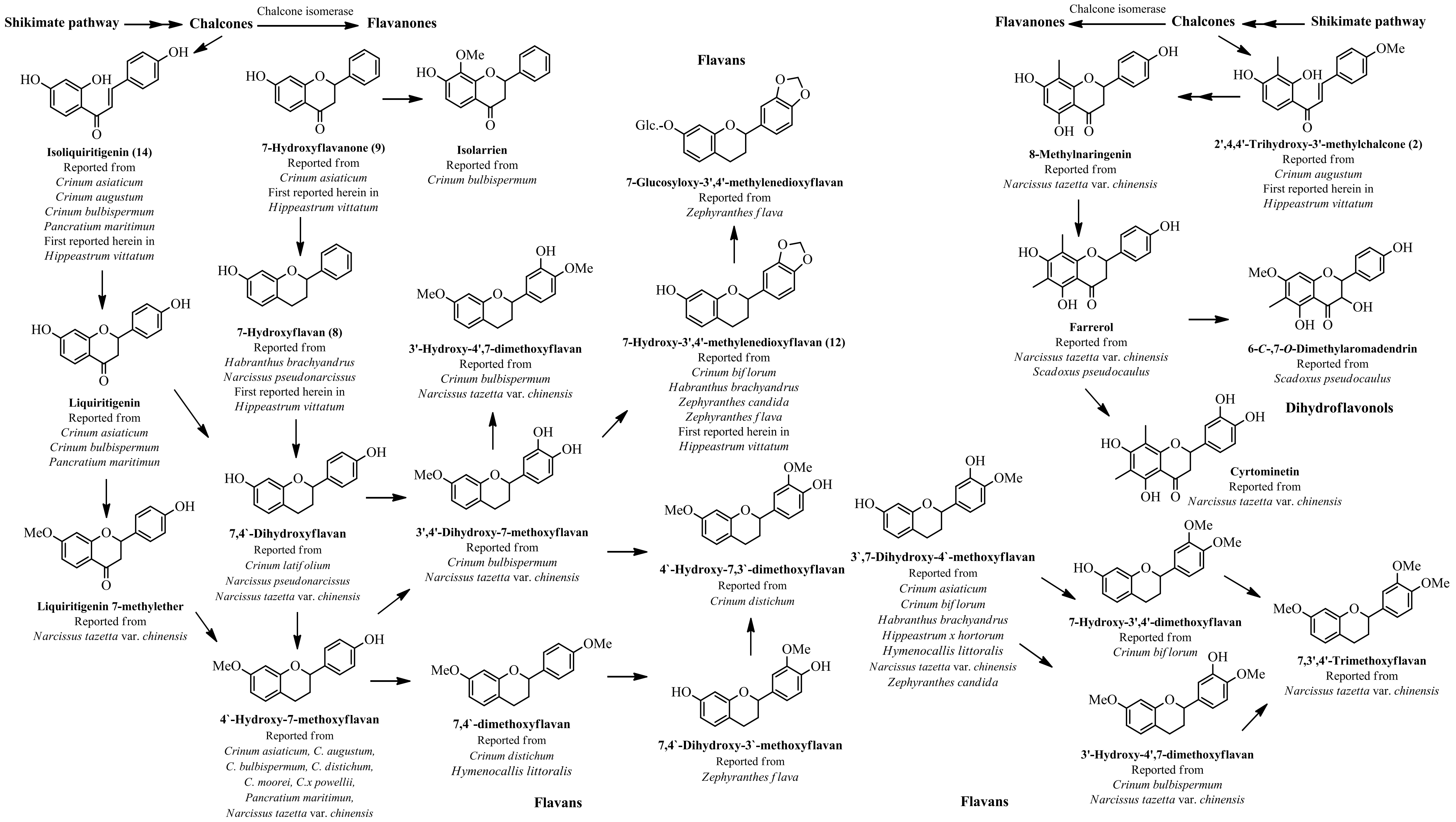

Scheme S3. Plausible biosynthetic pathway of flavanones and flavans from *H. vittatum* in comparison with those reported from other Amaryllidaceae plants.

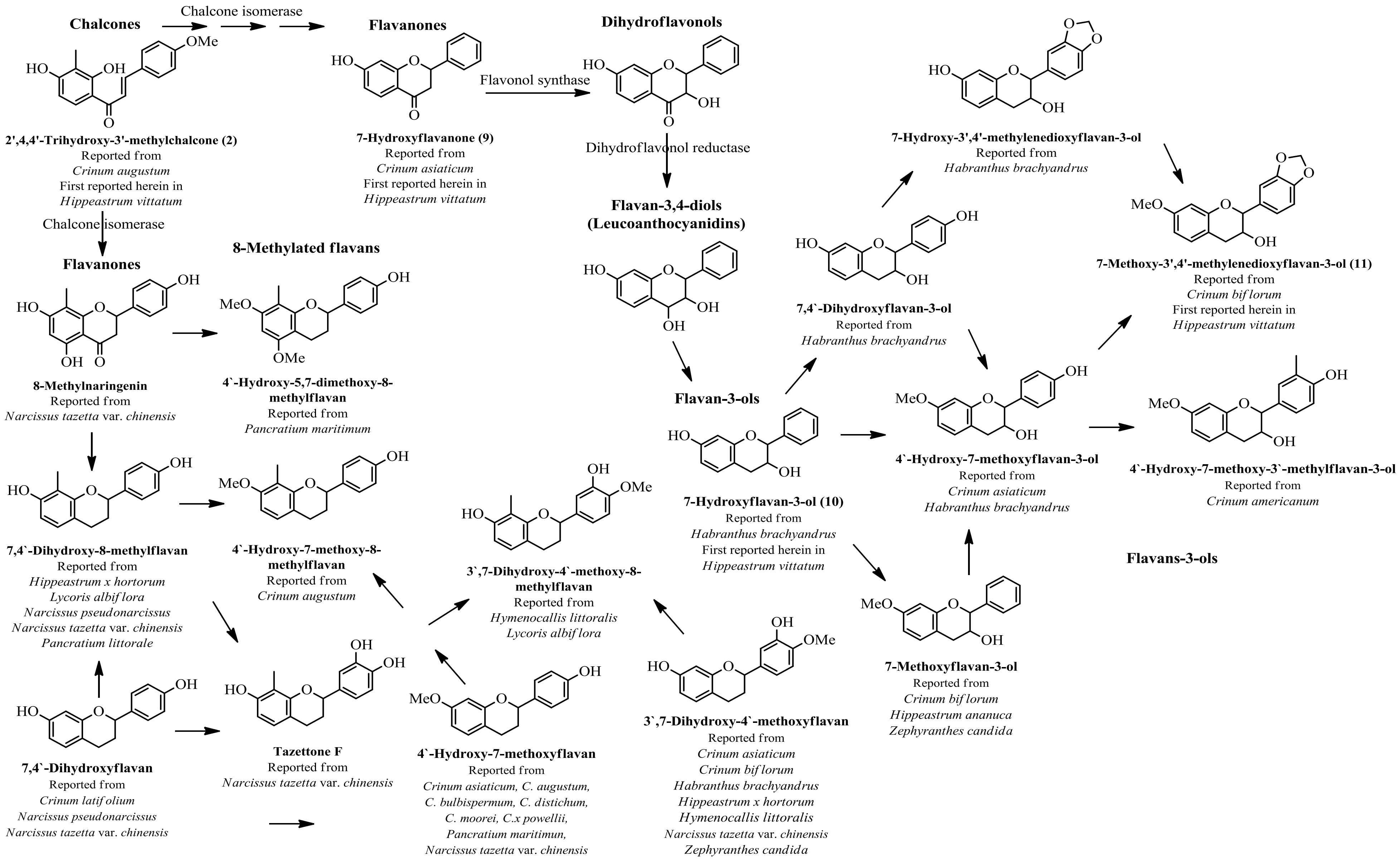

Scheme S4. Plausible biosynthetic pathway of 8-methylated flavans and flavan-3-ols from *H. vittatum* in comparison with those reported from other Amaryllidaceae plants.

II) Molecular docking analysis of the isolated compounds 1–16 from *H. vittatum* bulbs against SARS-CoV-2 proteins

Table S1. Energy scores (kcal/mol) and types of interactions of compounds **1–16**, compared to N3, with different amino acid residues in the binding site of SARS-CoV-2 M^{pro}.

Compound	Energy score (S) (kcal/mol)	Interactions		
		Amino acid residue	Bond type	Bond length (Å)
Lycorine **(1)**	–5.41	MET 165<br>CYS 145<br>GLU 166	H-bond<br>H-bond<br>Pi-H	3.72<br>3.25<br>4.18
2',4,4'-Trihydroxy-3'-methylchalcone (3'-methyl isoliquiritigenin) **(2)**	–6.05	MET 165<br>PHE 140	H-bond<br>H-bond	3.25<br>2.78
2,6-Dimethoxy-4-hydroxyacetophenone **(3)**	–5.13	THR 190<br>MET 165	H-bond<br>H-bond	2.90<br>3.78
2,4-Dihydroxyacetophenone **(4)**	–4.60	ARG 188<br>CYS 145<br>MET 165	H-bond<br>H-bond<br>Pi-H	2.87<br>4.18<br>3.67
p-Nitrophenol **(5)**	–4.35	CYS 145	H-bond	3.38
2,4-Dihydroxy-6-methoxy-3-methylacetophenone **(6)**	–4.88	MET 165<br>GLU 166<br>GLN 189	H-bond<br>Pi-H<br>Pi-H	3.24<br>4.27<br>4.34
2,4,6-Trimethoxyacetophenone **(7)**	–5.38	MET 165<br>GLU 166	H-bond<br>Pi-H	4.00<br>4.21
7-Hydroxyflavan **(8)**	–5.49	LEU 141<br>GLU 166	H-bond<br>Pi-H	3.16<br>4.40
7-Hydroxyflavanone **(9)**	–5.50	GLU 166<br>GLN 189	H-bond<br>Pi-H	3.18<br>3.85
7-Hydroxyflavan-3-ol **(10)**	–5.27	THR 190	H-bond	2.96
7-Methoxy-3',4'-methylenedioxyflavan-3-ol **(11)**	–6.04	LEU 141<br>GLU 166	H-bond<br>Pi-H	3.18<br>4.38
7-Hydroxy-3',4'-methylenedioxyflavan **(12)**	–5.75	LEU 141<br>GLU 166	H-bond<br>Pi-H	3.16<br>4.39
2',4'-Dihydroxy-3'-methyl-3,4-methylenedioxychalcone **(13)**	–6.11	CYS 145<br>CYS 44	H-bond<br>H-bond	3.04<br>3.53

2',4,4'-Trihydroxychalcone (isoliquiritigenin) **(14)**	–5.73	MET 165<br>PHE 140<br>ARG 188<br>THR 190	H-bond<br>H-bond<br>H-bond<br>H-bond	3.38<br>2.90<br>3.29<br>2.85
Narciprimine **(15)**	–5.98	GLN 102<br>MET 165<br>GLU 166<br>GLU 166<br>GLN 189	H-bond<br>Pi-H<br>Pi-H<br>Pi-H<br>Pi-H	3.09<br>3.96<br>3.81<br>4.27<br>3.77
β-Sitosterol 3-*O*-*β*-glucopyranoside **(16)**	**–8.28**	GLU 166<br>MET 165<br>MET 165<br>HIS 41	H-bond<br>H-bond<br>H-bond<br>H-Pi	3.19<br>3.55<br>3.86<br>4.49
N3	**–8.30**	CYS 145<br>MET 165<br>MET 165<br>GLY 143<br>GLN 189<br>GLN 189	H-bond<br>H-bond<br>H-bond<br>H-bond<br>Pi-H<br>Pi-H	3.79<br>3.74<br>3.48<br>3.06<br>3.67<br>4.08

Table S2. Energy scores (kcal/mol) and types of interactions of compounds **1–16**, compared to VIR251, with different amino acid residues in the binding site of SARS-CoV-2 PLpro.

**Compound**	**Energy score (S) (kcal/mol)**	**Interactions**		
		**Amino acid residue**	**Bond type**	**Bond length (Å)**
Lycorine **(1)**	–5.15	GLU 167<br>LYS 157	H-bond<br>H-bond	2.83<br>2.96
2',4,4'-Trihydroxy-3'-methylchalcone (3'-methyl isoliquiritigenin) **(2)**	–5.54	–	–	–
2,6-Dimethoxy-4-hydroxyacetophenone **(3)**	–5.19	MET 208<br>ARG 166<br>PRO 247	H-bond<br>H-bond<br>Pi-H	3.66<br>3.09<br>4.70

2,4-Dihydroxyacetophenone **(4)**	–4.68	TYR 273<br>TYR 264<br>PRO 247	H-bond<br>H-Pi<br>Pi-H	2.90<br>3.99<br>4.43
p-Nitrophenol **(5)**	–4.51	ASP 302<br>ASP 302<br>TYR 273	H-bond<br>H-bond<br>H-bond	3.27<br>3.07<br>2.91
2,4-Dihydroxy-6-methoxy-3-methylacetophenone **(6)**	–4.91	TYR 301<br>MET 208	H-bond<br>H-bond	2.89<br>3.83
2,4,6-Trimethoxyacetophenone **(7)**	–5.82	ARG 166<br>TYR 264<br>PRO 247	H-bond<br>H-Pi<br>Pi-H	2.91<br>4.16<br>4.52
7-Hydroxyflavan **(8)**	–5.52	ASP 302	H-bond	3.42
7-Hydroxyflavanone **(9)**	–4.75	TYR 264	Pi-Pi	3.83
7-Hydroxyflavan-3-ol **(10)**	–5.06	ASP 164	H-bond	2.92
7-Methoxy-3',4'-methylenedioxyflavan-3-ol **(11)**	–5.45	TYR 268<br>GLN 269	H-bond<br>Pi-H	3.13<br>4.20
7-Hydroxy-3',4'-methylenedioxyflavan **(12)**	–5.16	GLN 269	Pi-H	4.18
2',4'-Dihydroxy-3'-methyl-3,4-methylenedioxychalcone **(13)**	–5.68	GLN 269	H-bond	3.29
2',4,4'-Trihydroxychalcone (isoliquiritigenin) **(14)**	–5.27	–	–	–
Narciprimine **(15)**	–4.74	THR 301	H-bond	2.98
β-Sitosterol 3-*O*-*β*-glucopyranoside **(16)**	**–7.59**	ASP 164<br>ASP 164<br>ASP 302<br>ARG 166	H-bond<br>H-bond<br>H-bond<br>H-bond	3.38<br>3.16<br>3.12<br>2.94
VIR251	**–7.85**	TYR 273<br>GLY 163<br>TYR 268<br>ASP 164<br>GLY 271<br>TYR 268<br>ASP 164<br>LEU 162	H-bond<br>H-bond<br>H-bond<br>H-bond<br>H-bond<br>H-bond<br>Ionic<br>Pi-H	3.15<br>3.05<br>3.14<br>3.02<br>2.91<br>2.95<br>3.02<br>3.95

Table S3. Energy scores (kcal/mol) and types of interactions of compounds **1–16**, compared to remdesivir, with different amino acid residues in the binding site of SARS-CoV-2 RdRp.

**Compound**	**Energy score (S) (kcal/mol)**	**Interactions**		
		**Amino acid residue**	**Bond type**	**Bond length (Å)**
Lycorine **(1)**	–5.47	ASP 623	H-bond	2.69
		ARG 553	H-bond	3.10
		Mg 1004	Metal	2.21
2',4,4'-Trihydroxy-3'-methylchalcone (3'-methyl isoliquiritigenin) **(2)**	–5.88	ASP 760	H-bond	2.83
		MET 542	H-bond	4.43
		ARG 553	H-bond	3.21
		ARG 553	H-bond	3.17
		SER 682	Pi-H	4.23
2,6-Dimethoxy-4-hydroxyacetophenone **(3)**	–5.51	ARG 553	H-bond	2.99
		Mg 1005	Matal	2.08
2,4-Dihydroxyacetophenone **(4)**	–5.36	ASP 760	H-bond	2.74
		Mg 1004	Metal	2.07
p-Nitrophenol **(5)**	–4.69	THR 680	H-bond	3.08
		ARG 624	H-bond	3.01
		SER 682	Pi-H	3.78
2,4-Dihydroxy-6-methoxy-3-methylacetophenone **(6)**	–5.81	ARG 555	H-bond	2.94
2,4,6-Trimethoxyacetophenone **(7)**	–5.64	ARG 555	H-bond	3.09
7-Hydroxyflavan **(8)**	–5.52	SER 682	Pi-H	4.00
7-Hydroxyflavanone **(9)**	–5.61	ARG 553	H-bond	2.92
		SER 682	Pi-H	4.07
7-Hydroxyflavan-3-ol **(10)**	–5.36	ARG 555	H-bond	2.99
		SER 682	Pi-H	3.75
7-Methoxy-3',4'-methylenedioxyflavan-3-ol **(11)**	–5.82	ARG 555	H-bond	3.02
7-Hydroxy-3',4'-methylenedioxyflavan **(12)**	–5.48	ARG 553	H-bond	3.20
		LYS 551	H-bond	3.34
2',4'-Dihydroxy-3'-methyl-3,4-methylenedioxychalcone **(13)**	–6.54	ASP 760	H-bond	2.80
		ARG 553	H-bond	3.11
		ARG 553	H-bond	3.33

2',4,4'-Trihydroxychalcone (isoliquiritigenin) **(14)**	-5.84	THR 556	H-bond	2.87
		THR 680	H-bond	3.21
		ASP 760	H-bond	2.80
Narciprimine **(15)**	–5.67	SER 682	Pi-H	4.25
		THR 680	H-bond	3.05
		ARG 624	H-bond	2.93
β-Sitosterol 3-*O*-*β*-glucopyranoside **(16)**	**–11.30**	TRP 617	H-bond	2.83
		ASP 760	H-bond	2.67
		GLU 811	H-Bond	3.16
		Mg 1005	Metal	1.98
		Mg 1004	Metal	2.17
		Mg 1005	Metal	2.05
		Mg 1005	Metal	2.57
Remdesivir	**–9.85**	ASP 760	H-bond	2.77
		ARG 553	H-bond	3.37
		ARG 553	H-bond	3.04
		Mg 1004	Metal	2.15
		Mg 1005	Metal	2.18
		Mg 1004	Metal	2.10
		Mg 1005	Metal	2.04
		Mg 1005	Ionic	2.18
		Mg 1005	Ionic	2.04
		ARG 555	Pi-cation	3.48

References

(1) El-Moghazi, A.M.; Ali, A.A.; Mesbah, M.K. Phytochemical investigation of *Hippeastrum vittatum* growing in Egypt. Part II. Isolation and identification of new alkaloids. *Planta Med.* **1975**, *28*, 336–340.

(2) De Andrade, J.P.; Pigni, N.B.; Torras Claveria, L.; Guo, Y.; Berkov, S.; Reyes-Chilpa, R.; el Amrani, A.; Zuanazzi, J.A.S.; Codina, C.; Viladomat, F. Alkaloids from the *Hippeastrum* genus: chemistry and biological activity. *Rev. Latinoam. Quim.* **2012**, *40*, 83–98.

(3) Abd El-Hafiz, M.A.; Ramadan, M.; Anton, R. Minor phenolic constituents of *Crinum augustum*. *J. Nat. Prod.* **1990**, *53*, 1349–1352.

(4) Youssef, D.T.; Ramadan, M.; Khalifa, A. Acetophenones, a chalcone, a chromone and flavonoids from *Pancratium maritimum*. *Phytochemistry* **1998**, *49*, 2579–2583.

(5) Li, J.; Kadota, S.; Kawata, Y.; Hattori, M.; Xu, G.-J.; Namba, T. Constituents of the roots of *Cynanchum bungei* Decne. Isolation and structures of four new glucosides, bunngeiside-A,-B,-C, and-D. *Chem. Pharm. Bull.* **1992**, *40*, 3133–3137.

(6) Sun, Y.; Liu, Z.; Wang, J.; Xiang, L.; Zhu, L. Separation and purification of baishouwubenzophenone, 4-hydroxyacetophenone and 2,4-dihydroxyacetophenone from *Cynanchum auriculatum* Royle ex Wight by HSCCC. *Chromatographia* **2009**, *70*, 1–6.

(7) Abraham, R.J.; Mobli, M. An NMR, IR and theoretical investigation of ^{1}H chemical shifts and hydrogen bonding in phenols. *Mag. Res. Chem.* **2007**, *45*, 865–877.

(8) Chiaradia, L.D.; Dos Santos, R.; Vitor, C.E.; Vieira, A.A.; Leal, P.C.; Nunes, R.J.; Calixto, J.B.; Yunes, R.A. Synthesis and pharmacological activity of chalcones derived from 2, 4, 6-trimethoxyacetophenone in RAW 264.7 cells stimulated by LPS: Quantitative structure activity relationships. *Bioorg. Med. Chem.* **2008**, *16*, 658–667.

(9) Coxon, D.T.; O'Neill, T.M.; Mansfield, J.W.; Porter, A.E. Identification of three hydroxyflavan phytoalexins from daffodil bulbs. *Phytochemistry* **1980**, *19*, 889–891.

(10) Jitsuno, M.; Yokosuka, A.; Sakagami, H.; Mimaki, Y. Chemical constituents of the bulbs of *Habranthus brachyandrus* and their cytotoxic activities. *Chem. Pharm. Bull.* **2009**, *57*, 1153–1157.

(11) De Oliveira, A.B.; Fonseka e Silva; L.G.; Gottlieb, O.R. Flavonoids and coumarins from *Platymiscium praecox*. *Phytochemistry* **1972**, *11*, 3515–3519.

(12) Ghosal, S.; Singh, S.K.; Srivastava, R.S. Flavans from *Zephyranthes flava*. *Phytochemistry* **1985**, *24*, 151–153.

(13) Ramadan, M.; Kamel, M.; Ohtani, K.; Kasai, R.; Yamasaki, K. Minor phenolics from *Crinum bulbispermum* bulbs. *Phytochemistry* **2000**, *54*, 891–896.

(14) Nair, J.J.; Aremu, A.O.; van Staden, J. Isolation of narciprimine from *Cyrtanthus contractus* (Amaryllidaceae) and evaluation of its acetylcholinesterase inhibitory activity. *J. Ethnopharmacol.* **2011**, *137*, 1102–1106.
